# Paternal Caffeine Exposure Programs Offspring Stress Vulnerability via Sperm Dlk1‐Dio3 Imprinting‐Directed Remodeling of a Novel Neural Circuit

**DOI:** 10.1002/advs.75380

**Published:** 2026-04-30

**Authors:** Mengxi Lu, Gaole Dai, Sen Zhu, Shuai Zhang, Tingting Wang, Yuan Meng, Fang Yang, Xiaoyi Han, Hui Wang, Hao Kou, Dan Xu

**Affiliations:** ^1^ Department of Obstetrics School of Pharmaceutical Sciences Zhongnan Hospital of Wuhan University Wuhan University Wuhan China; ^2^ Department of Pharmacology Taikang Medical School (School of Basic Medical Sciences) Wuhan University Wuhan China; ^3^ Hubei Provincial Key Laboratory of Developmentally Originated Disease Wuhan China; ^4^ Department of Pharmacy Zhongnan Hospital of Wuhan University Wuhan China; ^5^ Key Laboratory of Combinatorial Biosynthesis and Drug Discovery Ministry of Education China

**Keywords:** Dlk1‐Dio3 domain, HPA axis hyperresponsivity, imprinted miRNA cluster, paternal preconception caffeine exposure, vCA1^Glu^ → Pir^GABA^ → PVN^CRH^ circuit

## Abstract

Paternal environmental exposures program offspring neurodevelopment via sperm epigenetics, yet mechanisms for intergenerational hypothalamic–pituitary–adrenal (HPA) axis dysregulation, a core hub for stress disorders, remain elusive. Using a paternal preconception caffeine exposure (PPCE) rat model with in vitro fertilization to exclude maternal confounders, we uncover a novel pathway linking sperm epigenetics to offspring HPA axis hyperresponsivity. By elevating paternal corticosterone, PPCE induces hypomethylation at the intergenic differentially methylated region (IG‐DMR) within sperm Dlk1‐Dio3 domain. This epigenetic alteration evades postfertilization reprogramming, persists in offspring hippocampus, and derepresses the maternally expressed miRNA cluster, causing posttranscriptional downregulation of glutaminase (GLS). Hippocampal GLS deficiency impairs glutamatergic neurotransmission in a novel circuit: ventral hippocampal CA1 glutamatergic neurons (vCA1^Glu^) → piriform cortex γ‐aminobutyric acid‐ergic neurons (Pir^GABA^) → paraventricular nucleus corticotropin‐releasing hormone neurons (PVN^CRH^). Chemogenetic activation of this circuit rescues HPA axis hyperresponsivity and affective phenotypes. Clinically, sperm IG‐DMR hypomethylation correlates with elevated plasma cortisol in prospective fathers. Importantly, paternal folic acid supplementation prevents these epigenetic alterations and restores offspring stress homeostasis. Our study delineates an intergenerational mechanism and identifies a potentially translatable prenatal intervention strategy.

## Graphical abstract

The study elucidates that paternal preconception stress can drive offspring hyperresponsivity of the stress system via hypomethylation of a specific DNA region in sperm. This key link is confirmed in a cohort of prospective fathers: the epigenetic alteration is associated with elevated stress hormone levels. Importantly, paternal folic acid supplementation provides a feasible strategy to prevent this intergenerational risk.

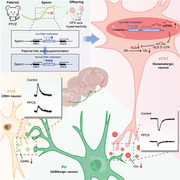

## 1 Introduction

The hypothalamic–pituitary–adrenal (HPA) axis serves as the core neuroendocrine hub for the body's stress response, and its homeostatic regulation is crucial for maintaining overall health [1]. By releasing glucocorticoids (cortisol in humans and corticosterone [CORT] in rodents), the HPA axis reallocates energy resources to cope with stress. The timely termination and precise regulation of this pathway are as important as its activation [2]. Excessive or sustained activation of the HPA axis constitutes a common pathological basis for various neuropsychiatric disorders (such as depression and anxiety) and metabolic diseases [3, 4, 5, 6, 7]. The “Developmental Origins of Health and Disease” (DOHaD) theory reveals that dysregulation of the HPA axis may originate from early‐life programming. Studies have demonstrated that when mothers are exposed to adverse factors such as stress during pregnancy, excessive glucocorticoids can cross the placenta and directly disrupt fetal HPA axis development, resulting in persistent dysregulation of stress responses in the offspring [8, 9, 10, 11]. In recent years, research attention has increasingly shifted toward the intergenerational effects of paternal environmental exposures. Both epidemiological and animal studies have shown significant associations between adverse paternal environments (such as nutritional imbalance or psychological stress) and offspring metabolic disorders as well as neurobehavioral abnormalities [12, 13, 14, 15, 16, 17]. However, whether and how paternal information specifically regulates the offspring's HPA axis—the central hub of stress response—remains a critical mechanistic question yet to be elucidated.

Long‐term exposure to adverse environmental factors (e.g., exogenous chemicals or psychological stress) promotes chronic stress and elevates disease susceptibility. As a central nervous system stimulant, caffeine intake induces sustained elevations in plasma cortisol levels, acting as a chronic stressor with documented reproductive and developmental toxicity [18, 19, 20, 21, 22]. Statistics from the International Coffee Organization indicate a rise in global caffeine consumption from 10.27 million tons (2018/19) to 10.62 million tons (2023/24), showcasing a widespread increase in population‐level exposure. Notably, large‐scale cohort studies indicate that reproductive‐aged men consume approximately 1.5‐fold higher caffeine than women, suggesting that the potential impact of paternal caffeine consumption on offspring health warrants close attention [23, 24]. Our prior work established that paternal preconception caffeine exposure (PPCE) disrupts offspring liver development and metabolic homeostasis, effects linked to elevated paternal glucocorticoid levels [25]. Given the critical role of glucocorticoids in gametogenesis and embryonic programming [26, 27, 28], this study investigates how PPCE and associated glucocorticoid alterations impact offspring HPA axis development.

The function of the HPA axis is precisely regulated by the limbic system, among which the hippocampus serves as a high‐level negative feedback center essential for maintaining HPA axis homeostasis [29]. The hippocampus sends glutamatergic projections to upstream nuclei of the paraventricular nucleus (PVN), such as the bed nucleus of the stria terminalis (BNST) and the medial preoptic area (mPOA), thereby exciting γ‐aminobutyric acid‐ergic (GABAergic) neurons within these nuclei. These GABAergic neurons, in turn, project to and inhibit the activity of the PVN, ultimately exerting a “long‐range inhibitory” effect on the HPA axis to terminate the stress response [30]. Our previous studies were the first to demonstrate that maternal caffeine exposure during pregnancy can reduce the activity of hippocampal glutamatergic neurons in the offspring, thereby weakening the negative feedback regulation of the HPA axis mediated by this long‐range inhibitory circuit. Consequently, the offspring exhibit hyperresponsivity of the HPA axis and increased susceptibility to neuropsychiatric and metabolic disorders [31, 32, 33, 34]. However, whether a similar neural pathway mediates the effects of paternal intergenerational programming, or whether alternative mechanisms are involved, remains to be determined.

Paternal preconception exposure to adverse environmental factors can mediate intergenerational inheritance by altering sperm epigenetic modifications, such as DNA methylation and noncoding RNAs [35, 36]. Among these, microRNAs (miRNAs), a class of key noncoding RNAs, participate in various cellular processes related to gametogenesis and embryonic development [37]. It has been demonstrated that chronic unpredictable stress in male mice significantly alters the miRNA expression profile in sperm, and these alterations are closely associated with depression‐like behaviors in the offspring [36]. The largest miRNA cluster in mammals is located within the Dlk1‐Dio3 imprinted domain. Multiple miRNAs within this cluster (such as miR‐134 and members of the miR‐379/410 cluster) are highly enriched in the brain and play essential roles in neurogenesis, synaptic plasticity, and neuronal migration through the regulation of target genes [38, 39, 40]. Imprinted genes exhibit parent‐of‐origin‐specific expression, meaning that only the allele inherited from either the mother or the father is expressed. This process is precisely regulated by imprinting control regions (ICRs), which are typically differentially methylated regions (DMRs) displaying opposite methylation states on maternal and paternal chromosomes. The intergenic differentially methylated region (IG‐DMR) serves as the core regulatory element governing gene expression within the Dlk1‐Dio3 imprinted domain. It is hypermethylated on the paternal chromosome and hypomethylated on the maternal chromosome [41]. This differential epigenetic marking directly mediates the maternal‐specific expression pattern of the miRNA cluster within this region. However, whether this precisely regulated imprinted domain can serve as an epigenetic carrier of paternal environmental exposure—particularly whether the IG‐DMR undergoes reprogramming and escapes postfertilization epigenetic remodeling following paternal caffeine exposure—remains unclear.

In this study, a PPCE rat model was established and combined with in vitro fertilization (IVF) technology to determine the independent programming effects of paternal environmental factors. The study yielded three key breakthrough findings. First, PPCE offspring exhibited hyperresponsivity of the HPA axis and anxiety‐ and depression‐like behaviors. This phenotype was specifically mediated by synaptic transmission impairment within a previously unrecognized neural circuit: ventral hippocampal CA1 glutamatergic neurons (vCA1^Glu^) → piriform GABAergic neurons (Pir^GABA^) → PVN corticotropin‐releasing hormone neurons (PVN^CRH^). Second, PPCE specifically induced hypomethylation of the IG‐DMR within the paternal Dlk1‐Dio3 imprinted domain. This epigenetic modification successfully escaped embryonic reprogramming and was stably transmitted to the offspring hippocampus, driving upregulation of the imprinted miRNA cluster, which in turn targeted and suppressed glutaminase (GLS) expression, ultimately impairing glutamatergic neurotransmission in the vCA1 region. Finally, through paternal glucocorticoid receptor (GR) antagonism experiments and in vitro spermatogonia models, it was confirmed that PPCE‐induced CORT‐mediated hypomethylation programming of the sperm IG‐DMR. These findings deepen our understanding of the mechanisms underlying paternal environmental exposure–induced intergenerational programming and provide a new theoretical framework for the origins of paternally derived neuropsychiatric phenotypes.

## 2 Results

### 2.1 PPCE Triggers HPA Axis Hyperresponsivity and Anxiety‐ and Depression‐Like Behaviors in Offspring Rats

**FIGURE 1:**
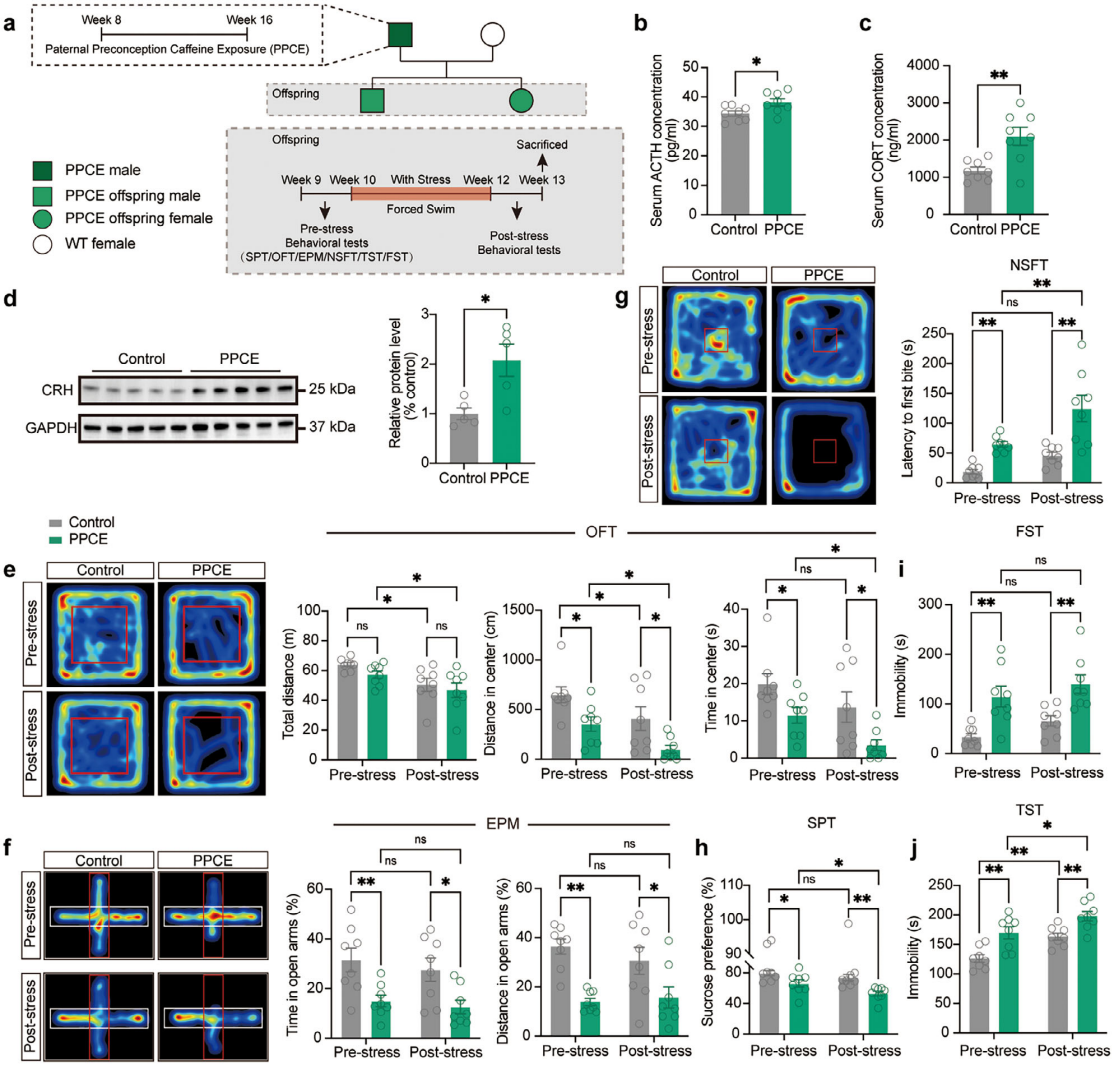
PPCE programs male offspring HPA axis hyperresponsivity and affective dysregulation. (a) Schematic diagram of animal experiments. (b) Serum ACTH levels in male PPCE offspring rats (*n* = 8 rats per group). (c) Serum CORT levels in male PPCE offspring rats (*n* = 8 rats per group). (d) CRH protein expression level in male PPCE offspring rats (*n* = 5 rats per group). (e) Schematic diagram of representative rat activity trajectories and statistical indicators in the OFT, including total distance, distance in center and time in center (*n* = 8 rats per group). (f) Schematic diagram of representative rat activity trajectories and statistical indicators in the EPM test, including time and distance spent in open arms (*n* = 8 rats per group). (g) Schematic diagram of representative rat activity trajectories and latency to first bite statistics in the NSFT (*n* = 8 rats per group). (h) Sucrose preference index in the SPT (*n* = 8 rats per group). (i) Immobility time in the FST (*n* = 8 rats per group). (j) Immobility time in the TST (*n* = 8 rats per group). Data are presented as mean ± SEM. ns, not significant; ^*^p < 0.05, ^**^p < 0.01; by unpaired two‐tailed *t*‐test (b–d), two‐way ANOVA (e–j).

To investigate PPCE effects on offspring HPA axis reactivity, we first measured baseline serum levels of adrenocorticotropic hormone (ACTH) and CORT in the offspring. Subsequently, the offspring rats were subjected to a 2‐week ice‐water swimming stress protocol. Immediately poststress, samples were collected to analyze serum ACTH and CORT levels alongside hypothalamic corticotropin‐releasing hormone (CRH) protein expression. Compared with controls, both male and female PPCE offspring exhibited significantly increased serum ACTH and CORT levels (Figure 1b,c and Figure S1a,b) and markedly upregulated hypothalamic CRH protein expression (Figure 1d and Figure S1c), collectively indicating HPA axis hyperresponsivity in PPCE offspring. Notably, although absolute hormone levels increased in both sexes after stress, analysis of the percent change relative to baseline revealed deeper regulatory differences. On one hand, the stress hormone gain rate was significantly higher in PPCE offspring compared to their sex‐matched controls. On the other hand, this growth effect exhibited sexual dimorphism: in the control group, the ACTH increase rate was significantly higher in female offspring than in male offspring (Figure S2a), consistent with previous reports that females generally exhibit stronger HPA axis reactivity [42, 43]. In contrast, in the PPCE group, no significant differences were observed between males and females in either ACTH or CORT increase rates (Figure S2a,b), suggesting that PPCE may enhance HPA axis responsiveness in male offspring, thereby abolishing the sex differences present under physiological conditions. These results indicate that PPCE not only broadly enhances HPA axis reactivity but also exerts a more pronounced effect on male offspring.

Given that HPA axis hyperresponsivity contributes to the pathogenesis of neurodevelopmental disorders [44, 45], we conducted serial behavioral tests before and after ice‐water swimming stress. Prestress assessments revealed: in the open field test (OFT), compared to the control group, male PPCE offspring showed no significant difference in total distance moved, but exhibited significant reductions in center distance and center duration (Figure 1e). In contrast, female PPCE offspring showed no significant changes in any of the measured parameters (Figure S1d). Elevated Plus Maze (EPM) testing showed significantly reduced open arm time and distance in male and female PPCE offspring (Figure 1f and Figure S1e). Novelty suppressed feeding test (NSFT) indicated significantly prolonged feeding latency in male and female PPCE offspring (Figure 1g and Figure S1f). These prestress results demonstrate anxiety‐like behaviors in male PPCE offspring independent of locomotor dysfunction, with females showing anxiety‐like tendencies. Sucrose preference test (SPT) showed significantly reduced sucrose preference in both sexes (Figure 1h and Figure S1g). Forced swim test (FST) and tail suspension test (TST) revealed significantly prolonged immobility time in males (Figure 1i,j), while females exhibited this prolongation only in the FST (Figure S1h) and not in the TST (Figure S1i), indicating depression‐like behaviors in male and female PPCE offspring. Poststress, both male and female PPCE offspring exhibited significant anxiety‐like and depression‐like behaviors (Figure 1e–j and Figure S1d–i). Collectively, these results demonstrate that PPCE induces anxiety‐ and depression‐like behaviors in offspring, exacerbated poststress with significant sexual dimorphism—males exhibiting more severe behavioral alterations. Therefore, based on the more severe dysregulation of both HPA axis reactivity and stress‐related behaviors observed in male offspring, all subsequent mechanistic investigations were focused on this group.

### 2.2 Inhibition of the vCA1^Glu^ → Pir^GABA^ → PVN^CRH^ Circuit Mediates HPA Axis Hyperresponsivity and Anxiety‐ and Depression‐Like Behaviors in PPCE Offspring

**FIGURE 2:**
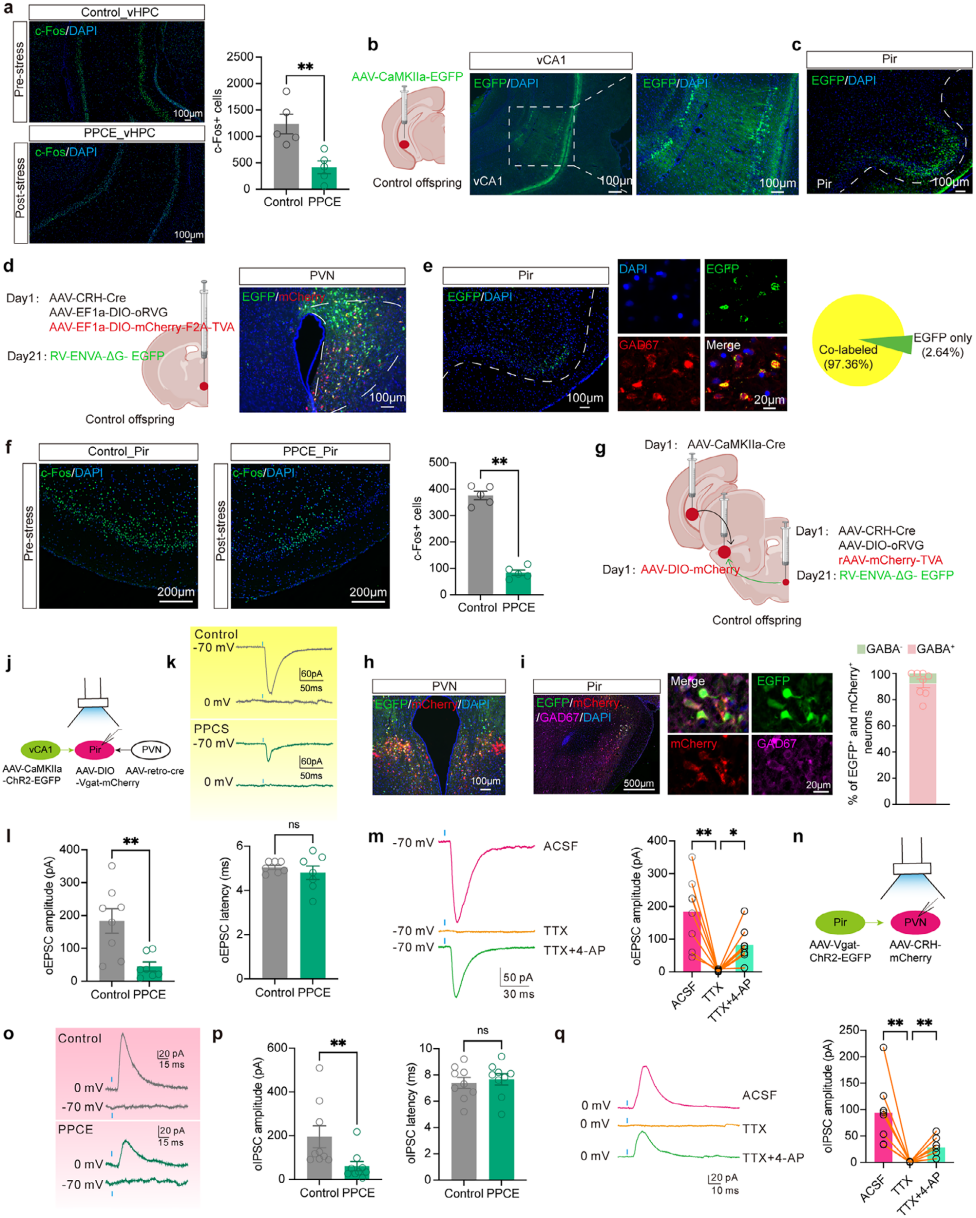
Impaired vCA1^Glu^ → Pir^GABA^ → PVN^CRH^ synaptic transmission in PPCE offspring. (a) c‐Fos immunofluorescence staining and positive cell count statistics (*n* = 5 rats per group). (b) Viral injection strategy and fluorescent representation of injection site. (c) Representative images of EGFP‐positive signals expressed in Pir. (d) Viral injection strategy and fluorescent representation of injection site. (e) Representative images of EGFP‐positive signal expression in Pir (left) and EGFP‐labeled neurons colocalized with GAD67 antibody within Pir (right). (f) c‐Fos immunofluorescence staining and positive cell count statistics (*n* = 5 rats per group). (g,h) Viral injection strategy and fluorescent representation of injection site. (i) Representative images of mCherry‐positive and EGFP‐positive signals in Pir (left), mCherry and EGFP double‐labeled neurons colocalized with GAD67 antibody (right). (j) Schematic of viral injection and the recording configuration. (k,l) Representative traces and summary data of oEPSCs recorded in the Pir, including oEPSCs latency and oEPSCs amplitude (*n* = 8 or 7 cells per group from 3 rats). (m) Example traces of postsynaptic responses of vCA1^Glu^ to Pir^GABA^ projections induced by optical stimuli (as indicated by blue ticks) under bath of artificial cerebrospinal fluid (ACSF), and in addition of TTX, TTX + 4‐AP (*n* = 8 cells per group from 3 rats). (n) Schematic of viral injection and the recording configuration. (o,p) Representative traces and summary data of oIPSCs recorded in the PVN, including oIPSCs latency and oIPSCs amplitude (*n* = 9 cells per group from 3 rats). (q) Example traces of postsynaptic responses of Pir^GABA^ to PVN^CRH^ projections induced by optical stimuli (as indicated by blue ticks) under bath of ACSF, and in addition of TTX, TTX + 4‐AP (*n* = 8 cells per group from 3 rats). Data are presented as mean ± SEM. ns, not significant; ^*^p < 0.05, ^**^p < 0.01; by unpaired two‐tailed *t*‐test (a, f, and p), Welch's *t* test (l), Mann–Whitney test (p), Friedman test (m) or one‐way ANOVA (q).

The function of the HPA axis is coordinately regulated by the hippocampus and amygdala. The hippocampus exerts negative feedback regulation on the HPA axis by activating GABAergic interneurons, facilitating the restoration of hyperactivated HPA axis to baseline levels, whereas the amygdala activates the HPA axis by inhibiting GABAergic inputs to the PVN [29]. c‐Fos immunofluorescence staining in poststress brain tissues revealed significantly reduced c‐Fos‐positive cells in ventral hippocampus (vHPC) and dorsal hippocampus (dHPC) in PPCE offspring versus controls (Figure 2a and Figure S3a,d), with no detectable changes in the amygdala (Figure S3b,d). This indicates suppressed hippocampal neuronal activity in PPCE offspring, potentially contributing to their HPA axis hyperresponsivity. Given that dHPC primarily modulates spatial learning/memory whereas vHPC—particularly its vCA1 subregion—serves as a critical hub for stress response and HPA axis regulation [46, 47], we focused subsequent investigations on the vCA1 area. Immunofluorescence for glutamatergic neuronal marker glutamate (Glu) and GABAergic marker glutamate decarboxylase 67 (GAD67) demonstrated significantly decreased Glu expression in PPCE offspring vCA1 at gestational day 20 (GD20), adult baseline (postnatal week 12, PW12), and post ice‐water stress, with unaltered GAD67 expression (Figure S4a–c). These findings preliminarily indicate specific and persistent impairment of vCA1 glutamatergic neuronal function in PPCE offspring starting from GD20.

Classical theories posit that vCA1 suppresses HPA axis activity through glutamatergic projections to GABAergic neurons in PVN‐projecting regions, including BNST, mPOA, anterior hypothalamic nuclei (AHN), dorsomedial hypothalamus (DMH), and ventromedial hypothalamus (VMH) [30]. However, our study revealed no significant intergroup differences in c‐Fos expression within these regions in PPCE offspring post ice‐water swimming stress (Figure S3c,d), suggesting vCA1 may regulate HPA axis via novel relay nuclei. Using an anterograde monosynaptic tracing strategy, we injected AAV‐CaMKIIα‐EGFP into vCA1 of control offspring (Figure 2b and Figure S3e). Three weeks later, EGFP‐positive neurons were detected in the Pir, lateral septum (LS), and anteroventral thalamic nucleus (ventrolateral, AVVL) (Figure 2c and Figure S3f). Subsequent pseudotyped rabies virus‐mediated retrograde monosynaptic tracing in control offspring—involving PVN coinjection of AAV‐CRH‐Cre (targeting CRH‐positive neurons) and Cre‐dependent helper viruses (AAV‐EF1α‐DIO‐mCherry‐F2A‐TVA + AAV‐EF1α‐DIO‐oRVG), followed by EnvA‐pseudotyped RV‐ΔG‐EGFP rabies virus (RV) at the same site 3 weeks later (Figure 2d)‐identified EGFP‐positive signals in Pir colocalized with GAD67 (Figure 2e). Critically, PPCE offspring exhibited significantly reduced Pir c‐Fos expression poststress (Figure 2f), indicating suppressed neuronal activity. Collectively, these findings identify Pir GABAergic neurons as the putative relay nucleus mediating vCA1^Glu^‐driven HPA axis inhibition.

To further validate the vCA1^Glu^ → Pir^GABA^ projection, we injected AAV‐CaMKIIα‐Cre into vCA1 and AAV‐DIO‐GAD67‐mCherry into Pir of control offspring (Figure S5a), observing mCherry‐positive fibers in Pir 3 weeks postinjection (Figure S5b). Subsequent Pir injection of AAV‐GAD67‐Cre (a GABA neuron‐targeted Cre recombinase) combined with rabies virus‐mediated monosynaptic retrograde tracing—entailing coinjection of Cre‐dependent helper viruses and RV—revealed EGFP‐positive neurons in vCA1 colocalized with Glu antibody (Figure S5c,d). Anterograde monosynaptic tracing via Pir injection of AAV‐mDIX‐EGFP (Figure S5e) demonstrated EGFP‐positive signals in PVN after 3 weeks (Figure S5f). To verify projection specificity, Pir injection of AAV‐mDIX‐Cre with concurrent PVN injection of AAV‐CRH‐DIO‐mCherry (Figure S5g) yielded mCherry‐positive signals in PVN (Figure S5h). For comprehensive visualization of the vCA1^Glu^ → Pir^GABA^ → PVN^CRH^ circuit connectivity, we implemented integrated anterograde trans‐monosynaptic tracing from vCA1 (AAV‐CaMKIIα‐Cre injection) to Pir (AAV‐DIO‐mCherry injection) and retrograde trans‐monosynaptic tracing targeting PVN CRH‐positive neurons (Figure 2g,h), detecting both mCherry‐positive and EGFP‐positive signals in Pir with partial double‐labeled neurons colocalized with GAD67 (Figure 2i), conclusively establishing the vCA1^Glu^ → Pir^GABA^ → PVN^CRH^ circuit.

To further characterize functional connectivity of the vCA1^Glu^ → Pir^GABA^ → PVN^CRH^ circuit, we employed channelrhodopsin‐2 (ChR2)‐assisted circuit mapping (CRACM). ChR2, a light‐gated ion channel, was expressed in vCA1^Glu^ neurons via AAV‐CaMKIIα‐ChR2‐EGFP injection into vCA1 of control offspring. Concurrent Pir injection of AAV‐DIO‐Vgat‐mCherry and PVN injection of AAV‐retro‐Cre to label PVN‐projecting Pir^GABA^ neurons (Figure 2j). Blue light stimulation of vCA1^Glu^ terminals in Pir elicited optogenetically‐induced excitatory postsynaptic currents (oEPSCs) within 10 ms in mCherry‐labeled Pir^GABA^ neurons (Figure 2k,l). These oEPSCs were abolished by tetrodotoxin (TTX, sodium channel blocker preventing action potentials) but restored with coapplication of 4‐aminopyridine (4‐AP, potassium channel antagonist enabling presynaptic depolarization) (Figure 2m), confirming monosynaptic vCA1^Glu^ → Pir^GABA^ connectivity. Repeating this in PPCE offspring revealed comparable oEPSC latency but significantly reduced amplitude versus controls (Figure 2l). Subsequent Pir injection of AAV‐Vgat‐ChR2‐EGFP (restricting ChR2 to Pir^GABA^ neurons) and PVN injection of AAV‐CRH‐mCherry (labeling PVN^CRH^ neurons) in both groups (Figure 2n) showed light activation of Pir^GABA^ axons in PVN‐induced sub‐10 ms optogenetically‐induced inhibitory postsynaptic currents (oIPSCs) in PVN^CRH^ neurons (Figure 2o,p). TTX abolished oIPSCs, while 4‐AP coapplication restored currents (Figure 2q). PPCE offspring exhibited unchanged oIPSC latency but significantly reduced amplitude versus controls (Figure 2p). Collectively, these results demonstrate intact monosynaptic connectivity in the PPCE offspring circuit but significantly impaired glutamatergic (vCA1 → Pir) and GABAergic (Pir → PVN) synaptic efficacy.

**FIGURE 3:**
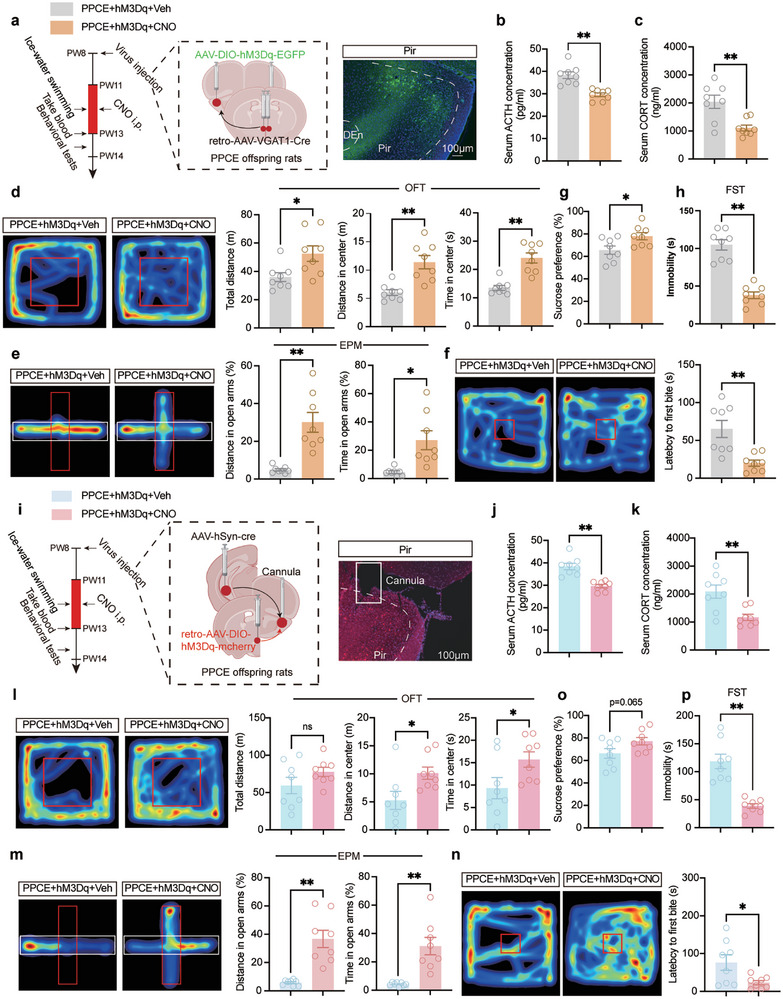
Circuit‐specific inhibition mediates HPA axis /behavioral deficits in PPCE offspring. (a) Schematic diagram of animal handling and fluorescent representation of injection sites. (b) Serum ACTH levels (*n* = 8 rats per group). (c) Serum CORT levels (*n* = 8 rats per group). (d) Schematic diagram of representative rat activity trajectories and statistical indicators in the OFT, including total distance, distance in center and time in center (*n* = 8 rats per group). (e) Schematic diagram of representative rat activity trajectories and statistical indicators in the EPM test, including time and distance spent in open arms (*n* = 8 rats per group). (f) Schematic diagram of representative rat activity trajectories and latency to first bite statistics in the NSFT (*n* = 8 rats per group). (g) Sucrose preference index in the SPT (*n* = 8 rats per group). (h) Immobility time in the FST (*n* = 8 rats per group). (i) Schematic diagram of animal handling and fluorescent representation of injection sites. (j) Serum ACTH levels (*n* = 8 rats per group). (k) Serum CORT levels (*n* = 8 rats per group). (l) Schematic diagram of representative rat activity trajectories and statistical indicators in the OFT, including total distance, distance in center and time in center (*n* = 8 rats per group). (m) Schematic diagram of representative rat activity trajectories and statistical indicators in the EPM test, including time and distance spent in open arms (*n* = 8 rats per group). (n) Schematic diagram of representative rat activity trajectories and latency to first bite statistics in the NSFT (*n* = 8 rats per group). (o) Sucrose preference index in the SPT (*n* = 8 rats per group). (p) Immobility time in the FST (*n* = 8 rats per group). Data are presented as mean ± SEM. ns, not significant; ^*^p < 0.05, ^**^p < 0.01; by unpaired two‐tailed *t*‐test (b–d, g, h, j–l, o), or Welch's *t* test (d, e, f, m, n, p).

To elucidate the pivotal role of Pir^GABA^ → PVN projections in mediating HPA axis hyperresponsivity in PPCE offspring, we injected retro‐AAV‐Vgat1‐Cre into PVN and AAV‐DIO‐hM3Dq‐EGFP into Pir of PPCE offspring. After 3 weeks for viral expression, rats underwent a 2‐week ice‐water swimming stress protocol with concurrent intraperitoneal clozapine N‐oxide (CNO) injections to selectively activate the Pir^GABA^ → PVN pathway (Figure 3a). Compared to vehicle‐treated controls, CNO‐activated PPCE offspring exhibited significantly reduced poststress serum ACTH and CORT (Figure 3b,c), increased center activity in OFT and open arm exploration in EPM (Figure 3d,e), improved feeding behavior in NSFT (Figure 3f), elevated sucrose preference (Figure 3g), and reduced immobility time in FST (​​Figure 3h), collectively demonstrating reversal of HPA axis hyperresponsivity and anxiety‐ and depression‐like behaviors. To further dissect vCA1 → Pir → PVN circuit regulation, we injected AAV‐hSyn‐Cre into vCA1 and retro‐AAV‐DIO‐hM3Dq‐mCherry into PVN of PPCE offspring, then intracranially infused CNO into Pir to activate vCA1‐innervated PVN‐projecting Pir neurons during stress (Figure 3i). Continuous CNO perfusion significantly attenuated HPA axis responses and anxiety‐ and depression‐like behaviors (Figure 3j–p). Collectively, these results establish a long‐range vCA1^Glu^ → Pir^GABA^ → PVN^CRH^ circuit whose suppression mediates PPCE‐induced HPA axis hyperresponsivity and affective behavioral deficits.

### 2.3 Suppressed GLS Expression in the vCA1 Region Mediates Functional Impairment of the vCA1^Glu^ → Pir^GABA^ → PVN^CRH^ Circuit in PPCE Offspring

**FIGURE 4:**
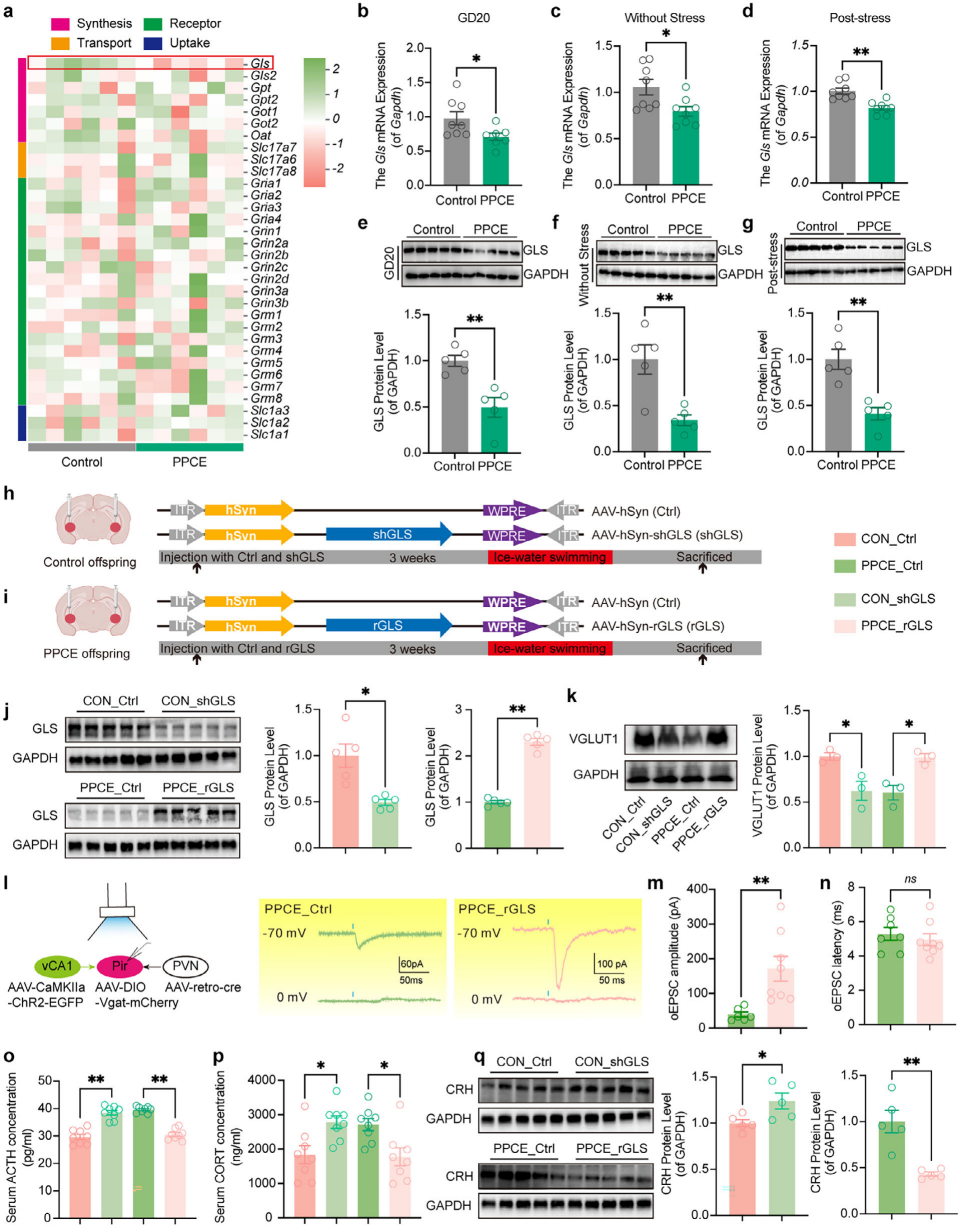
GLS deficiency drives HPA axis dysfunction via novel circuit suppression. (a) Heatmap of Glu system‐related gene expression in the vCA1 tissue of PPCE offspring rats. (b–d) Gls mRNA expression in the vCA1 tissue of PPCE offspring rats (*n* = 8 litters or rats per group). (e–g) GLS protein expression in the vCA1 tissue of PPCE offspring rats (*n* = 5 litters or rats per group). (h,i) Schematic diagram of viral injection and animal handling. (j) GLS protein expression level (*n* = 5 rats per group). (k) vGluT1 protein expression level (*n* = 3 rats per group). (l) Schematic of viral injection and the recording configuration, representative traces of oEPSCs recorded in the Pir. (m,n) Summary data of oEPSCs recorded in the Pir, including oEPSCs latency (*n* = 7 or 8 cells per group from 3 rats) and oEPSCs amplitude (*n* = 6 or 8 cells per group from 3 rats). (o) Serum ACTH concentration (*n* = 8 rats per group). (p) Serum CORT concentration (*n* = 8 rats per group). (q) CRH protein expression level (*n* = 5 rats per group). Data are presented as mean ± SEM. ns, not significant; ^*^p < 0.05, ^**^p < 0.01; by unpaired two‐tailed *t*‐test (b–g, j, m, n, and q), Welch's *t* test (j and q), or one‐way ANOVA (k, o, and p).

To investigate molecular mechanisms underlying weakened glutamatergic synaptic transmission in vCA1 neurons of PPCE offspring, we conducted transcriptome sequencing of vCA1 tissue. Glutamatergic synaptic function involves coordinated regulation of Glu synthesis, transport, receptor signaling, and reuptake: Glu generation depends on transamination of tricarboxylic acid cycle‐derived α‐ketoglutarate (α‐KG) or astrocyte‐derived glutamine catalyzed by GLS, followed by vesicular loading via vesicular glutamate transporters (vGluTs), synaptic release, activation of postsynaptic Glu receptors, and finally reuptake of residual Glu via excitatory amino acid transporters on astrocytes/presynaptic neurons. Transcriptomic analysis of Glu synapse‐related genes revealed significantly downregulated GLS expression—a key enzyme for Glu synthesis—with no alterations in α‐KG‐synthesizing transaminases or genes involved in Glu transport, receptor function, or reuptake (Figure 4a). Consistent downregulation of GLS mRNA and protein expression was observed in PPCE offspring vCA1 across developmental stages (GD 20, adult baseline, and post ice‐water stress; Figure 4b–g), accompanied by reduced Glu content (Figure S6a–c) but unaltered α‐KG levels (Figure S6d–f). Collectively, these results suggest that GLS inhibition compromises presynaptic Glu biosynthesis in vCA1, thereby attenuating glutamatergic neurotransmission in PPCE offspring.

To establish the causal role of vCA1 GLS in HPA axis hyperresponsivity and anxiety‐ and depression‐like behaviors, we performed stereotaxic injections of neuron‐specific AAV‐hSyn‐shGLS into vCA1 of control offspring to knock down GLS (CON_shGLS), with AAV‐hSyn‐EGFP‐injected controls as counterparts (CON_Ctrl), while PPCE offspring received AAV‐hSyn‐rGLS for GLS overexpression (PPCE_rGLS) versus AAV‐hSyn‐EGFP‐injected PPCE controls (PPCE_Ctrl) (Figure 4h,i). After 3 weeks of viral expression followed by a 2‐week ice‐water swimming stress protocol, behavioral assessments and postmortem analysis confirmed successful GLS manipulation (Figure 4j). Poststress, CON_shGLS rats exhibited significantly reduced vGluT1 protein (glutamatergic neuron marker) in vCA1 versus CON_Ctrl (Figure 4k), concomitant with HPA axis hyperresponsivity—elevated serum ACTH and CORT (Figure 4o,p) and increased hypothalamic CRH protein (Figure 4q). Behaviorally, CON_shGLS showed decreased center activity in OFT (​​Figure S6i) and open arm exploration in EPM (​​Figure S6j), prolonged feeding latency in NSFT (Figure S6k), reduced sucrose preference (Figure S6m), and extended immobility time in FST (​​Figure S6l), indicating anxiety‐ and depression‐like phenotypes. Conversely, PPCE_rGLS significantly reversed HPA axis dysfunction and affective behaviors versus PPCE_Ctrl (Figure 4o–q). Employing CRACM in PPCE_rGLS rats—with vCA1 injection of AAV‐CaMKIIα‐ChR2‐EGFP, Pir injection of AAV‐DIO‐Vgat‐mCherry, and PVN injection of AAV‐retro‐Cre (Figure 4l)—blue light stimulation of vCA1^Glu^ terminals in Pir elicited oEPSCs in Pir^GABA^ neurons within 10 ms, showing unchanged latency but significantly increased amplitude versus PPCE_Ctrl (Figure 4m,n), confirming restored glutamatergic synaptic transmission. These findings demonstrate that vCA1 GLS downregulation is a pivotal molecular mechanism underlying PPCE‐induced pathologies, and targeted GLS elevation effectively rescues disease phenotypes.

### 2.4 Upregulation of the Dlk1‐Dio3 Imprinted Domain miRNA Cluster Targets and Suppresses GLS Expression in the vCA1 Region of PPCE Offspring

As critical noncoding RNAs, miRNAs extensively regulate nervous system development [48, 49], with prior studies indicating paternal separation stress elevates offspring brain miRNA expression to mediate memory deficits [50]. We examined miRNA profiles in PPCE offspring vCA1 tissue and identified consistent upregulation of 54 miRNAs within the Dlk1‐Dio3 imprinted domain—the largest miRNA cluster in mammals (Figure S7a). Integrated TargetScan and miRWalk database predictions revealed 35 miRNAs potentially targeting GLS. KEGG enrichment analysis of their targets showed 18 miRNAs significantly associated with neurotransmitter signaling and synaptic plasticity pathways. Based on expression abundance and fold‐change, we prioritized 8 core miRNAs. RT‐qPCR confirmed significant upregulation of these miRNAs in PPCE offspring vCA1 at GD20, adult baseline, and poststress (Figure S7b–d). To validate GLS targeting, we constructed dual‐luciferase reporters containing wild‐type (WT) or mutant (MUT) GLS 3′‐UTR sequences transfected into H19‐7 cells. miRNA mimics significantly reduced luciferase activity in WT 3′‐UTR groups versus controls but not in MUT groups (Figure S8a–h), confirming these miRNAs suppress GLS via specific 3′‐UTR binding. Collectively, these results demonstrate persistent activation of the Dlk1‐Dio3 miRNA cluster in PPCE offspring vCA1 mediates targeted GLS suppression.

**FIGURE 5:**
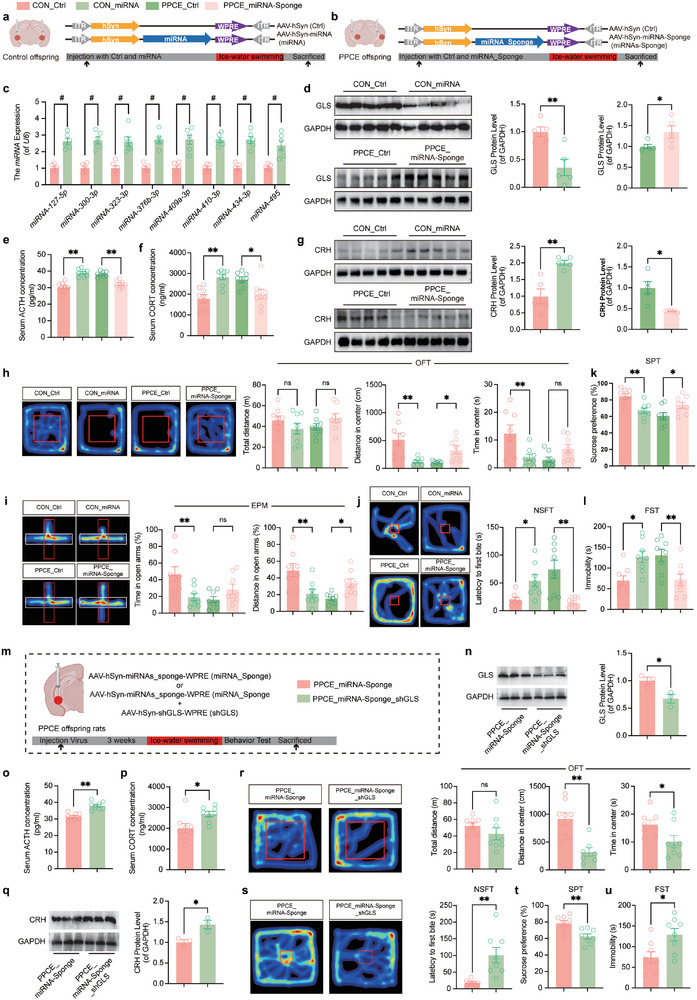
Dlk1‐Dio3 miRNA cluster targets GLS to program stress pathologies. (a,b) Schematic diagram of viral injection and animal handling. (c) Validation of AAV‐hSyn‐miRNA expression (*n* = 6 rats per group). (d) GLS protein expression level (*n* = 6 rats per group). (e) Serum ACTH concentration (*n* = 8 rats per group). (f) Serum CORT concentration (*n* = 8 rats per group). (g) CRH protein expression level (*n* = 6 rats per group). (h) Schematic diagram of representative rat activity trajectories and statistical indicators in the OFT, including total distance, distance in center and time in center (*n* = 8 rats per group). (i) Schematic diagram of representative rat activity trajectories and statistical indicators in the EPM test, including time and distance spent in open arms (*n* = 8 rats per group). (j) Schematic diagram of representative rat activity trajectories and latency to first bite statistics in the NSFT (*n* = 8 rats per group). (k) Sucrose preference index in the SPT (*n* = 8 rats per group). (l) Immobility time in the FST (*n* = 8 rats per group). (m) Schematic diagram of viral injection and animal handling. (n) GLS protein expression level (*n* = 3 rats per group). (o) Serum ACTH concentration (*n* = 8 rats per group). (p) Serum CORT concentration (*n* = 8 rats per group). (q) CRH protein expression level (*n* = 3 rats per group). (r) Schematic diagram of representative rat activity trajectories and statistical indicators in the OFT, including total distance, distance in center and time in center (*n* = 7 or 8 rats per group). (s) Schematic diagram of representative rat activity trajectories and latency to first bite statistics in the NSFT (*n* = 8 rats per group). (t) Sucrose preference index in the SPT (*n* = 8 rats per group). (u) Immobility time in the FST (*n* = 8 rats per group). Data are presented as mean ± SEM. ns, not significant; *^#^q* < 0.01, ^*^p < 0.05, ^**^p < 0.01; by multiple unpaired *t*‐test (c), unpaired two‐tailed *t*‐test (d, g, and n–u), Welch's *t* test (g), or one‐way ANOVA (e, f, and h–l).

To confirm the regulatory role of upregulated imprinted miRNAs in HPA axis hyperresponsivity, we constructed neuron‐specific AAV‐hSyn‐miRNA overexpression vectors targeting 8 core miRNAs. Comparing stereotaxic injection of AAV‐hSyn‐miRNA into vCA1 of control offspring (CON_miRNA) with injection of AAV‐hSyn‐EGFP (CON_Ctrl) validated miRNA upregulation (Figure 5a,c). After 3‐week viral expression and 2‐week ice‐water stress, CON_miRNA rats showed significantly reduced vCA1 GLS protein versus CON_Ctrl (Figure 5d), confirming miRNA targeting. Enzyme‐linked immunosorbent assay (ELISA) revealed elevated serum ACTH and CORT (Figure 5e,f) and increased hypothalamic CRH protein (​​Figure 5g) in CON_miRNA, indicating HPA axis hyperresponsivity. Behavioral tests poststress demonstrated anxiety‐like phenotypes (reduced OFT center activity and EPM open arm exploration, prolonged feeding latency; Figure 5h–j) and depression‐like behaviors (decreased sucrose preference and prolonged FST immobility; Figure 5k,l). Conversely, vCA1 injection of neuron‐specific AAV‐hSyn‐miRNA‐sponge in PPCE offspring (PPCE_miRNA‐Sponge) versus PPCE_Ctrl increased GLS protein (Figure 5b,d), attenuating HPA axis stress responses (Figure 5e–g) and anxiety‐ and depression‐like behaviors (Figure 5h–l). Further, coinjection of AAV‐hSyn‐miRNA‐sponge and AAV‐hSyn‐shGLS into PPCE offspring vCA1 (PPCE_miRNA‐Sponge_shGLS; Figure 5m,n) abolished the miRNA knockdown rescue, reinstating HPA axis hyperresponsivity and affective behaviors versus PPCE_miRNA‐Sponge (Figure 5o–u), demonstrating that GLS targeting is essential for miRNA‐mediated pathologies. Thus, persistent Dlk1‐Dio3 miRNA cluster overexpression in PPCE offspring vCA1 suppresses GLS, driving HPA axis hyperresponsivity and anxiety‐ and depression‐like behaviors.

### 2.5 Paternal Folic Acid (FA) Supplementation Rescues PPCE‐Induced Epigenetic Programming Abnormalities through Reversal of Sperm IG‐DMR Hypomethylation

**FIGURE 6:**
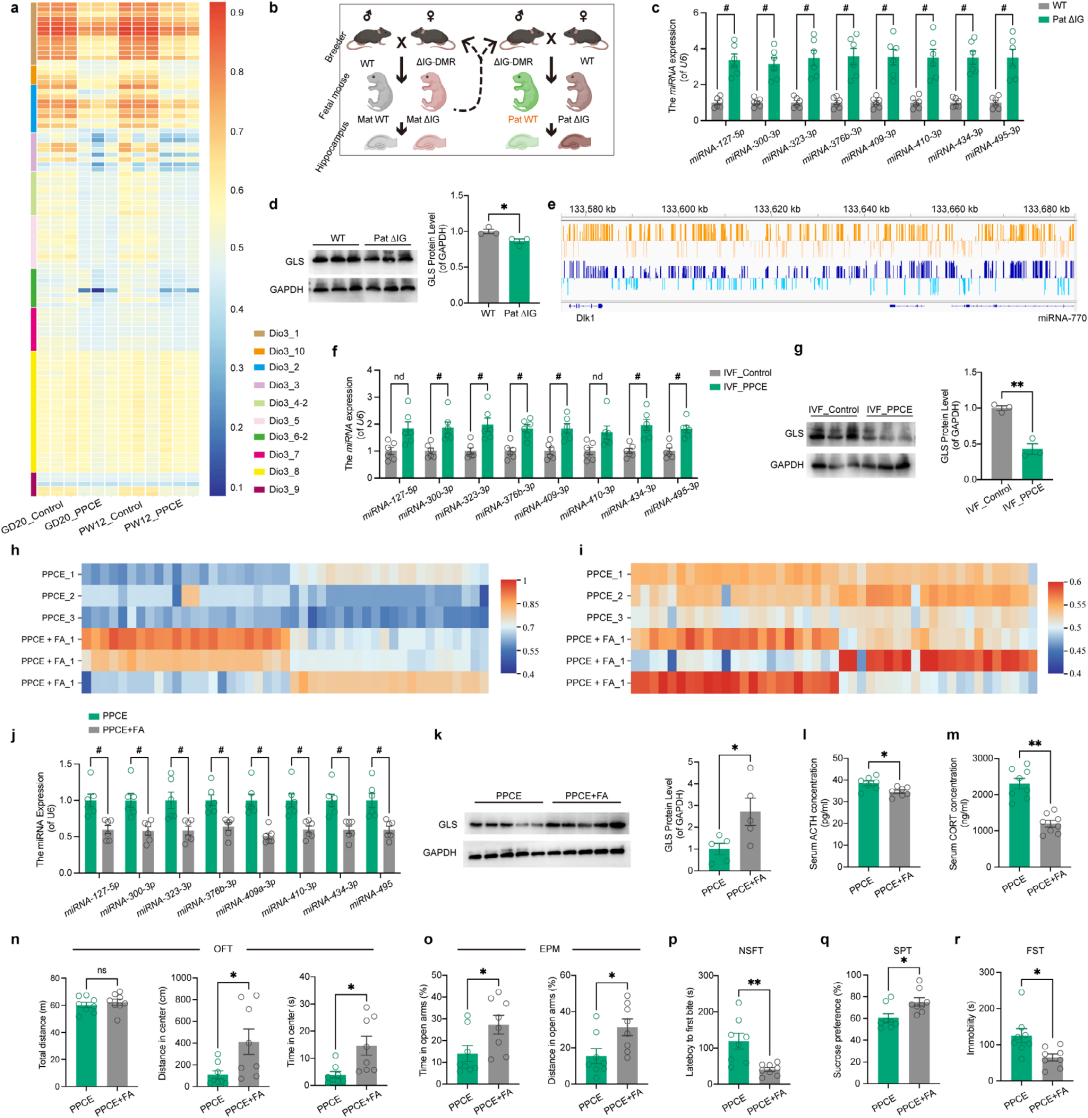
Sperm IG‐DMR hypomethylation intergenerationally upregulates hippocampal miRNAs. (a) BSP analysis of IG‐DMR methylation in vCA1 tissue of PPCE offspring rats (*n* = 3 rats per group). (b) Breeding schematic of paternal IG‐DMR knockout mice. (c) miRNA expression in Pat ΔIG mice (*n* = 6 rats per group). (d) GLS protein expression in Pat ΔIG mice (*n* = 3 rats per group). (e) Visualize WGBS data using IGV. (f) miRNA expression in IVF_PPCE offspring mice (*n* = 6 rats per group). (g) GLS protein expression in IVF_PPCE offspring mice (*n* = 3 rats per group). (h) BSP analysis of IG‐DMR methylation in sperm of paternal FA supplementation offspring rats (*n* = 3 rats per group). (i) BSP analysis of IG‐DMR methylation in vCA1 tissue of paternal FA supplementation offspring rats (*n* = 3 rats per group). (j) miRNA expression in paternal FA supplementation offspring rats (*n* = 6 rats per group). (k) GLS protein expression in paternal FA supplementation offspring rats (*n* = 5 rats per group). (l) Serum ACTH concentration in paternal FA supplementation offspring rats (*n* = 8 rats per group). (m) Serum CORT concentration in paternal FA supplementation offspring rats (*n* = 8 rats per group). (*n*) Statistical indicators in the OFT, including total distance, distance in center and time in center (*n* = 8 rats per group). (o) Statistical indicators in the EPM test, including time and distance spent in open arms (*n* = 8 rats per group). (p) Latency to first bite statistics in the NSFT (*n* = 8 rats per group). (q) Sucrose preference index in the SPT (*n* = 8 rats per group). (r) Immobility time in the FST (*n* = 8 rats per group). Data are presented as mean ± SEM. nd, no discovery; *^#^q* < 0.01, ^*^p < 0.05, ^**^p < 0.01; by multiple unpaired *t*‐test (c, f, and j), unpaired two‐tailed *t*‐test (d, g, k–o, q, and r) or Welch's *t* test (n and p). [Correction added on 27 May 2026, after first online publication: Figure 6 is updated in this version.]

Expression of the Dlk1‐Dio3 miRNA cluster is strictly regulated by parent‐of‐origin‐specific methylation patterns at IG‐DMR. To investigate whether PPCE disrupts this epigenetic regulation, we analyzed IG‐DMR methylation dynamics in PPCE offspring vCA1 using bisulfite sequencing PCR (BSP). Results revealed significantly reduced IG‐DMR methylation in PPCE offspring at fetal stage (GD20), persisting into adulthood (PW12) versus controls (Figure 6a). IG‐DMR comprises a bidirectional regulatory element with a 5′ IG^CGI^ (CpG island) and 3′ IG^TRE^ (transcriptional regulatory element). Crucially, the IG^CGI^ contains a 216‐bp tandem repeat sequence (IG‐DMR‐Rep)—identified as the key regulatory determinant for allele‐specific expression, independent of other IG‐DMR sites. Using a paternal chromosome‐specific IG‐DMR‐Rep knockout mouse model (Pat ΔIG; Figure 6b), we observed significantly upregulated miRNAs (​​Figure 6c) and downregulated GLS (Figure 6d) in fetal vCA1 versus WT. Collectively, these findings demonstrate that PPCE‐induced paternal IG‐DMR hypomethylation mediates persistent offspring vCA1 miRNA cluster overexpression and subsequent GLS suppression.

Sperm, as critical vectors carrying specific molecular and epigenetic modifications, constitute the molecular nexus linking paternal environmental exposures to offspring phenotypic traits [51, 52, 53, 54]. Studies indicate paternal trauma and stress adversely affect offspring via sperm epigenetic marks [55, 56]. Whole‐genome methylation sequencing (WGBS) revealed significant hypomethylation at the Dlk1‐Dio3 imprinted domain IG‐DMR in PPCE paternal sperm (Figure 6e). To establish causality between paternal sperm epigenetics and offspring pathology, we performed IVF using PPCE sperm (IVF_PPCE). IVF_PPCE offspring exhibited significantly upregulated vCA1 miRNA expression (Figure 6f) and downregulated GLS expression (​​Figure 6g), demonstrating that PPCE paternal sperm can independently induce the suppression of GLS expression in the offspring's vCA1 region, independent of maternal factors.

As a key methyl donor, FA plays a central role in regulating DNA methylation. Animal studies have confirmed that paternal FA deficiency can induce abnormal sperm DNA methylation and increase offspring disease risk [57], whereas paternal FA supplementation can mediate intergenerational enhancement of offspring axonal regeneration by inducing transgenerationally stable differential methylation modifications in sperm [58]. Based on this, we investigated whether paternal FA supplementation could correct the aberrant epigenetic programming induced by PPCE. The results demonstrated that FA supplementation effectively reversed the PPCE‐induced hypomethylation at the sperm IG‐DMR, and this methylation recovery was maintained in the vCA1 region of F1 offspring (Figure 6h,i). Concomitantly, the supplementation blocked the PPCE‐triggered overexpression of the Dlk1‐Dio3 miRNA cluster, thereby rescuing hippocampal GLS expression (Figure 6j,k). This series of molecular improvements ultimately manifested as a significant amelioration of offspring HPA axis stress reactivity and anxiety‐ and depression‐like behaviors (Figure 6l–r).

In summary, this study not only elucidates a novel mechanism by which PPCE programs offspring HPA axis hyper‑responsivity through sperm IG‑DMR hypomethylation but also demonstrates in an animal model that paternal FA intervention at clinically relevant doses can effectively reverse this aberrant programming.

### 2.6 Elevated CORT, Rather Than Caffeine Itself, Mediates PPCE‐Induced Hypomethylation of the IG‐DMR in Paternal Sperm

**FIGURE 7:**
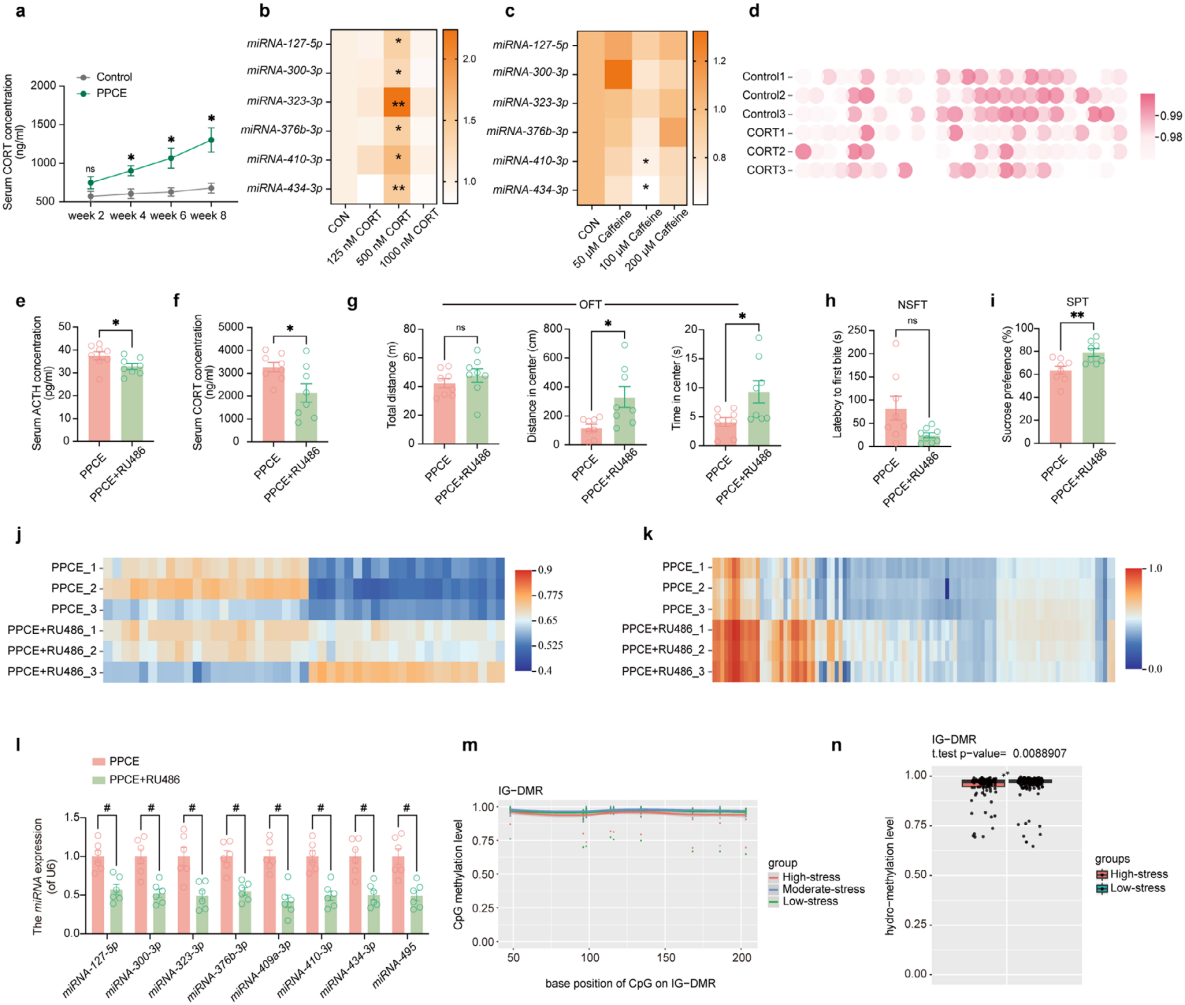
Elevated CORT mediates sperm IG‐DMR hypomethylation. (a) Serum CORT levels in paternal rats (*n* = 8 rats per group). (b) miRNA expression in GC‐1 cells after treatment with different concentrations of CORT (*n* = 6). (c) miRNA expression in GC‐1 cells after treatment with different concentrations of caffeine (*n* = 6). (d) BSP analysis of IG‐DMR methylation in GC‐1 cells treated with 500 nm CORT (*n* = 6). (e) Serum ACTH levels in RU486‐treated PPCE offspring rats (*n* = 8 rats per group). (f) Serum CORT levels in RU486‐treated PPCE offspring rats (*n* = 8 rats per group). (g) Statistical indicators in the OFT, including total distance, distance in center and time in center (*n* = 8 rats per group). (h) Latency to first bite statistics in the NSFT (*n* = 8 rats per group). (i) Sucrose preference index in the SPT (*n* = 8 rats per group). (j) BSP analysis of sperm IG‐DMR methylation in RU486‐treated paternal rats (*n* = 3 rats per group). (k) BSP analysis of IG‐DMR methylation in vCA1 tissue of RU486‐treated PPCE offspring rats. (l) miRNA expression in vCA1 tissue of RU486‐treated PPCE offspring rats (*n* = 6 rats per group). (m,n) Methylation level of the IG‐DMR in the Dlk1‐Dio3 imprinted domain in human sperm. Data are presented as mean ± SEM. nd, no discovery; *^#^q* < 0.01, ^*^p < 0.05, ^**^p < 0.01; by two‐way ANOVA (a), one‐way ANOVA (b, c), unpaired two‐tailed *t*‐test (e, f, i), multiple unpaired *t*‐test (k), or Welch's *t* test (g, h).

Caffeine, as a nonspecific adenosine receptor antagonist, has been shown in previous studies to induce anxiety‐ and depression‐like behaviors at high doses by antagonizing central A2A receptors [59]. Additionally, it exerts developmental toxicity by elevating plasma CORT levels. We first measured the serum CORT levels in paternal rats, and the results showed a sustained significant increase starting from week 4 (Figure 7a), indicating the successful establishment of a stable chronic stress environment induced by caffeine. To investigate whether the epigenetic changes in sperm induced by PPCE were directly mediated by caffeine or indirectly induced by the elevated CORT levels, we treated the mouse spermatogonia cell line GC‐1 with varying concentrations of caffeine (0–200 µm) or CORT (0–1000 nm). The results showed that 500 nm CORT treatment significantly induced IG‐DMR hypomethylation, accompanied by upregulation of miRNA cluster expression (Figure 7b,d). In contrast, 100 µm caffeine treatment actually reduced miRNA cluster expression levels to some extent (Figure 7c). These results suggest that caffeine and CORT have fundamentally different effects on the epigenetic regulation of spermatogonia cells, further confirming that CORT, rather than caffeine itself, is the key mediator of the epigenetic disturbances in germ cells in the PPCE model.

To further validate this pathway in vivo, we concurrently administered the GR‐specific antagonist mifepristone (RU486) via gavage to PPCE paternal rats and subjected their offspring to a 2‐week ice‐water swimming stress protocol. The results demonstrated that ​​paternal coadministration of RU486 significantly reversed HPA axis hyperresponsivity in PPCE offspring​​, as evidenced by markedly reduced serum ACTH and CORT levels following ice‐water swimming stress compared to the PPCE group (Figure 7e,f). Moreover, a battery of behavioral tests demonstrated significant alleviation of anxiety‐ and depression‐like phenotypes in PPCE offspring, as evidenced by increased exploratory activity in the center zone​​ of the OFT (Figure 7g) and markedly elevated sucrose preference index​​ in the SPT (Figure 7i). Consistent with these phenotypic improvements, RU486 intervention reversed both paternal sperm and offspring vCA1 IG‐DMR hypomethylation (Figure 7j,k) and normalized miRNA cluster expression in the offspring hippocampus (Figure 7l). These results collectively confirm that PPCE, through high levels of CORT activation of the GR signaling pathway, drives sperm IG‐DMR hypomethylation and subsequent intergenerational inheritance effects.

Finally, we validated the clinical relevance of this mechanism in a human cohort. We recruited 70 prospective fathers, systematically collecting peripheral blood and semen samples. Based on ​​peripheral blood cortisol concentration—a key biomarker of clinical stress levels​​—participants were stratified into three groups: low‐stress controls (cortisol < 200 µg/L), moderate‐stress (200–400 µg/L), and high‐stress (>400 µg/L). BSP showed significantly reduced sperm IG‐DMR methylation in high‐stress versus low‐stress men (Figure 7l,m), with no change in moderate‐stress group.

In summary, through triple validation using an in vitro spermatogonia cell model, in vivo pharmacological blockade, and clinical human cohort studies, we confirm that PPCE induces elevated CORT levels, rather than directly affecting germ cells, to drive epigenetic reprogramming of the sperm Dlk1‐Dio3 imprinted domain, thereby mediating its intergenerational inheritance effects.

## 3 Discussion

This study establishes a novel mechanistic axis for PPCE programming offspring HPA axis function via sperm epigenetics: PPCE induces hypomethylation at the IG‐DMR within the sperm Dlk1‐Dio3 imprinted domain through elevated CORT levels. This epigenetic alteration escapes postfertilization reprogramming, driving persistent overexpression of the mammalian largest miRNA cluster in offspring hippocampus, which targets and suppresses GLS synthesis. Critically, hippocampal GLS deficiency selectively impairs synaptic transmission in a novel neural circuit—​​vCA1^Glu^ → Pir^GABA^ → PVN^CRH^—ultimately triggering offspring HPA axis hyperresponsivity and anxiety‐ and depression‐like behaviors. Validated by IVF experiments, this reveals a “sperm epigenome‐offspring brain axis” for paternal environmental programming independent of maternal factors. Importantly, this pathogenic axis is reversible, concurrent paternal FA supplementation during PPCE prevented IG‐DMR hypomethylation and rescued downstream molecular events and behavioral phenotypes, suggesting the potential for targeted epigenetic intervention.

The caffeine dose employed in this study (60 mg/kg/day) warrants further contextualization within both real‐world exposure scenarios and experimental model requirements. Caffeine exhibits a well‐established biphasic dose‐response profile: low‐to‐moderate doses (50–400 mg in humans) enhance cognition and mood [60], whereas excessive intake (>400 mg) induces anxiety‐ and depression‐like behaviors and heightens stress responsivity [61]. This dose‐dependent dichotomy provided the rationale for selecting a high‐dose regimen to establish a paternal chronic stress model. Based on the body surface area conversion factor between humans and rats (1:6.17), the 60 mg/kg/day dose administered to rats corresponds to approximately 576 mg of caffeine per day for an adult male, equivalent to 4–6 cups of brewed coffee [62]. Although this exceeds the recommended upper limit for healthy adults [63], epidemiological data indicate that daily intakes at or above this level are not uncommon [23, 64, 65]. Therefore, rather than representing an extreme setting divorced from reality, this dosage was selected to simulate the long‐term high‐dose caffeine intake status observed in certain human subpopulations, carrying clear public health implications.

A key finding of this study is that the intergenerational effects of PPCE are not mediated by direct action of caffeine on germ cells, but rather by a chronic paternal stress state triggered by caffeine, characterized by sustained elevation of serum CORT. While our data strongly support CORT/GR signaling as the central mechanism underlying sperm IG‐DMR reprogramming, we cannot exclude the possibility that caffeine may exert additional effects on the sperm epigenome through other pathways, such as modulation of neurotransmitter systems (e.g., dopamine or adenosine signaling) [66], which warrant further investigation. Moreover, we acknowledge that high‐dose caffeine could potentially influence other physiological systems—including cardiovascular function, sleep patterns, metabolism, and nutrient absorption—that might independently affect HPA axis activity [65, 67, 68]. Although our findings point to CORT/GR signaling as a predominant pathway, the potential contributions of these other systems cannot be completely excluded based on the current data. Future studies incorporating comprehensive physiological monitoring would help dissect the relative contributions of these different pathways to the observed intergenerational effects. Mechanistically, the PPCE model can thus be established as an effective chemically induced stress model. This model provides a novel and mechanistically well‐defined research paradigm for investigating how common environmental substances can generate intergenerational effects through chronic endocrine stress pathways. By linking a widely consumed psychoactive substance to “paternal stress”, this study broadens the understanding of how environmental factors may impact reproductive health via stress pathways, suggesting that any lifestyle or environmental factors capable of chronically disrupting HPA axis homeostasis (such as other stimulants, circadian rhythm disruption, or chronic low‐grade inflammation) may pose similar intergenerational risks.

At the circuit level, we identified the vCA1^Glu^ → Pir^GABA^ → PVN^CRH^ circuit​​ as a previously unrecognized core hub for HPA axis regulation. Through anterograde/retrograde viral tracing, we confirmed vCA1^Glu^ neurons directly synapse onto Pir^GABA^ interneurons, which subsequently project to PVN^CRH^ neurons. Chemogenetic activation of any circuit node (Pir^GABA^ → PVN or vCA1 → Pir → PVN) rescued HPA axis/behavioral phenotypes, establishing the Pir as a novel relay nucleus within the HPA regulatory network that mediates hippocampal negative feedback. The Pir is a primary olfactory cortex and a key component of the limbic system, responsible for processing olfactory information and integrating it with emotion and memory. Kondoh et al. previously reported that the amygdalo‐piriform transition area, as a subregion of the olfactory cortex, transmits predator odor signals to PVN CRH neurons, thereby rapidly activating the HPA axis and inducing stress hormone release [69]. This finding revealed the anatomical basis for direct olfactory regulation of the HPA axis. The present study, by incorporating the Pir itself into the hippocampal‐mediated negative feedback network of the HPA axis, suggests that this brain region may play a dual role in both the initiation and termination of stress responses. Classical models have established that the hippocampus indirectly regulates the PVN through the BNST and multiple hypothalamic nuclei (e.g., mPOA, AHN, DMH, VMH) [30]. Compared to the classical BNST/hypothalamus‐mediated pathways, the vCA1^Glu^ → Pir^GABA^ → PVN^CRH^ pathway identified here may operate in parallel, collectively constituting a multilayered regulatory network for HPA axis control. In our PPCE offspring model, the classical pathways (BNST and hypothalamic nuclei) showed no significant changes in c‐Fos expression poststress, whereas the Pir pathway was markedly suppressed, indicating that the Pir pathway is more susceptible to PPCE‐induced developmental programming. The precise functional relationship between these two pathways—whether they operate independently, synergistically, or are recruited under different stress contexts—requires further investigation. This circuit discovery critically relates to clinical stress disorders: epilepsy patients exhibit Pir^GABA^ neuron loss and HPA axis dysregulation [70, 71], and our work first establishes its causal link in vivo.

At the mechanistic level, this study uncovers the complete pathway of intergenerational transmission from paternal stress to offspring phenotypes. We first identified a key epigenetic target, the Dlk1‐Dio3 imprinted domain, which undergoes hypomethylation following PPCE. This region encodes the largest miRNA cluster in mammals (containing 57 miRNAs). At the physiological level, the female‐biased high expression pattern of this cluster aligns with the heightened reactivity of the female HPA axis, suggesting a potential functional coupling between them. To establish the functional sufficiency of this epigenetic alteration, we employed a paternal chromosome‐specific IG‐DMR‐Rep knockout mouse model. This genetic manipulation alone was sufficient to recapitulate the core molecular phenotypes observed in PPCE offspring, specifically driving hippocampal miRNA upregulation with consequent GLS suppression. Having established its pivotal role, we then sought to trace the upstream signals responsible for reprogramming this locus. At the cellular origin, spermatogonia experiments revealed that CORT—not caffeine—directly induces IG‐DMR hypomethylation and activates the miRNA cluster. The centrality of the CORT‐GR axis was further validated *i*n vivo: pharmacological blockade of GR signaling by RU486 significantly reversed the intergenerational effects of PPCE. To conclusively isolate the paternal germline contribution, IVF using sperm from PPCE fathers was sufficient to induce the key molecular signatures in the absence of maternal confounders. More importantly, clinical data revealed that elevated cortisol levels in preconception males were significantly associated with sperm IG‐DMR hypomethylation, providing direct human evidence supporting the “CORT‐driven sperm epigenetic reprogramming” mechanism revealed by the animal models. In summary, this study integrates cellular, animal, and clinical evidence to elucidate, for the first time, the complete pathway by which PPCE induces CORT elevation, drives sperm IG‐DMR reprogramming, and leads to offspring HPA axis hyperresponsivity. These findings offer new insights into the intergenerational epigenetic mechanisms of paternal stress and deepen the “parental programming” theoretical framework in the DOHaD.

Several limitations of this study warrant attention: First, although both in vivo GR antagonism experiments and in vitro spermatogonia cell models have confirmed the central regulatory role of GC‐GR signaling in the methylation status of sperm IG‐DMR, the precise molecular mechanism of GC‐GR signaling‐mediated methylation regulation during spermatogenesis remains unclear. Second, while the IG‐DMR‐Rep knockout mouse model provided genetic evidence supporting the functional role of this imprinted region, it should be noted that these mice represent a whole‐body knockout, which cannot exclude potential contributions from IG‐DMR deletion in other tissues during development. Future studies utilizing region‐ and cell type‐specific conditional knockout models would help pinpoint the precise role of this imprinted domain in regulating offspring stress phenotypes. Moreover, direct intervention (e.g., targeted methylation editing) in PPCE sperm or offspring hippocampus to causally link IG‐DMR methylation to phenotypes is currently lacking. Third, offspring behavioral phenotypes exhibit significant sexual dimorphism, yet sex‐specific regulatory mechanisms are unexplored. Whether the core epigenetic mechanism (IG‐DMR hypomethylation → miRNA upregulation → GLS suppression) operates similarly in females, and how sex‐specific factors such as hormonal milieu modulate the downstream consequences, remain important questions for future investigation. Fourth, several limitations of the clinical cohort should be acknowledged. The modest sample size and cross‐sectional design preclude causal inference. Moreover, while we excluded individuals with major medical conditions and self‐reported smoking/alcohol/medication use, other potential confounders (e.g., diet, sleep, occupational stress, psychosocial factors) were not systematically assessed. Therefore, the observed association should be interpreted as preliminary correlational evidence requiring validation in larger, prospective cohorts.

Our proposed ​​Paternal Origins of Health and Disease (POHaD)​​ concept expands the DOHaD framework to include paternal environmental dimensions. Building upon the resistance to reprogramming exhibited by the IG‐DMR in the Dlk1‐Dio3 imprinted domain, we postulate a novel hypothesis: paternal stress may potentially influence methylation homeostasis at other imprinted domains through similar epigenetic anchoring mechanisms, consequently programming offspring susceptibility to both psychiatric disorders and metabolic diseases. Future research should focus on integrating the intergenerational regulatory network of “paternal epigenetic marks—offspring neuro‐metabolic axes” to identify novel therapeutic targets for interrupting the intergenerational transmission of psychiatric and metabolic disorders.

## 4 Materials and Methods

### 4.1 Experimental Animals and Treatments

Specific Pathogen Free (SPF)‐grade male Wistar rats (5 weeks old, GemPharmatech, China) were acclimated for 1 week under standard conditions (temperature 18–22°C, humidity 40–60%, 12 h light/dark cycle) prior to PPCE modeling. All animal experimental procedures followed the review and approval of the Institutional Animal Care and Use Committee of the Center for Animal Experiment at Wuhan University (Permit No. JC2020‐015). Given caffeine's biphasic dose‐response—low‐to‐moderate doses (50–400 mg) enhanced cognition and mood, whereas excessive intake induced anxiety‐ and depression‐like behaviors and hyperresponsivity [61]—the PPCE model was established using high‐dose caffeine. Experimental rats received daily oral gavage of 60 mg/kg caffeine (equivalent to 576 mg/day in adult males based on human‐rat dose conversion factor 6.17) for 8 weeks (spanning a full spermatogenic cycle), while controls received equal‐volume saline. Sperm from PPCE and control fathers underwent WGBS. For breeding, 2 females and 1 male were caged nightly at 18:00; vaginal smear examination the next morning confirmed GD0 upon sperm detection. Subsets of dams were sacrificed at GD20 for fetal collection, while others delivered naturally. Offspring were raised under standard conditions until PW10, then subjected to 2‐week ice‐water swimming stress (2–4°C, 5 min/day). Blood was collected via orbital sinus pre‐/poststress, followed by behavioral tests. A subgroup was maintained until PW12 without stress. Terminal procedures involved anesthesia with 2% isoflurane, euthanasia, and stereomicroscopic brain dissection for morphological/molecular analyses.

Additionally, PPCE paternal rats were randomly allocated to either the PPCE group or the RU486 intervention group (PPCE + 1 mg/kg/day RU486 via gavage). After 8 weeks of continuous intervention, males from both groups were bred with normal females. Resulting offspring were raised under standard conditions until PW10, then subjected to a 2‐week ice‐water swimming stress protocol. Blood samples were collected pre‐ and poststress, followed by a battery of behavioral tests upon stress completion.

Based on the recommended daily intake of FA for adults (400 µg/day) by the National Institutes of Health, the corresponding supplementation dose for rats was calculated as 41.35 µg/kg/day. Accordingly, male rats were randomly divided into two groups: the PPCE group and the FA intervention group (PPCE + 41.35 µg/kg/day FA via gavage). After 8 consecutive weeks of intervention, male rats from each group were mated with healthy female rats. The offspring were housed under standard conditions until PW10, followed by a 2‑week ice‑water swimming stress protocol. Samples were collected after stress for subsequent analyses.

### 4.2 IVF

Male C57BL/6 mice were randomly assigned to control and PPCE groups (*n* = 12 per group). The PPCE cohort received daily oral gavage of caffeine (120 mg/kg/day) for 8 consecutive weeks, while controls were administered equal volumes of saline. Postintervention, spermatozoa were collected via epididymal puncture and resuspended in human tubal fluid medium at a concentration of 2 × 10^6^/mL. Following a 15 min sperm activation period, subsequent procedures were initiated. Female C57BL/6 mice (*n* = 24, aged 6–8 weeks) underwent superovulation induction via intraperitoneal injection of 5 IU equine chorionic gonadotropin and 5 IU human chorionic gonadotropin. Mature oocytes were subsequently harvested from oviductal ampullae. Control or PPCE‐derived spermatozoa were coincubated with oocytes in embryo culture medium for fertilization. Resultant zygotes underwent 48 h in vitro culture. Viable embryos were surgically transferred into the oviducts of pseudopregnant recipient females. On GD20 post‐transfer, recipient dams were anesthetized with 2% isoflurane and subjected to cesarean section for fetal retrieval and sex determination. Fetal brain tissues were microdissected under stereomicroscopic guidance for morphological and molecular analyses. All sperm extraction and IVF procedures were executed by Cyagen Biosciences Co., Ltd (China).

### 4.3 Construction of IG‐DMR Knockout Mice

The IG‐DMR knockout mouse model (C57BL/6J‐IG‐DMR^em1Smoc^) was generated by Shanghai Model Organisms Center (China). Paternal knockout of the high‐CG‐repeat region in IG‐DMR resulted in postnatal lethality in most offspring. In contrast, maternal knockout permitted postnatal survival and breeding viability. The breeding strategy illustrated in Figure 6b was therefore implemented: offspring with paternal chromosome IG‐DMR knockout (IG‐DMR^+/ΔRep^) were utilized for experiments, while maternal chromosome knockout offspring (IG‐DMR^Δ/+Rep^) served for colony expansion. Embryos from crosses between IG‐DMR^Δ/+Rep^ females and WT males were dissected at GD20. Tail biopsies were genotyped, and Pat ΔIG embryos were selected for subsequent analyses.

### 4.4 Cell Culture and Treatment

Mouse spermatogonial cell line GC‐1 was obtained from the American Type Culture Collection (ATCC, USA). Cells were maintained in Dulbecco's modified Eagle's medium (DMEM; Gibco, USA) supplemented with 10% fetal bovine serum (Gibco, USA) at 37°C. To investigate the specific roles of CORT and caffeine, GC‐1 cells were treated with varying concentrations of CORT (0, 125, 500, 1000 nm) or caffeine (0, 50, 100, 200 µm) for 24 h, respectively. Following treatment, cells were harvested for analysis of miRNA cluster expression levels and DNA methylation.

### 4.5 Human Plasma and Sperm Analysis​​

Paired plasma and semen samples from 70 male participants were obtained through the Reproductive Medicine Department at Zhongnan Hospital, Wuhan University. Plasma cortisol concentrations and sperm IG‐DMR methylation rates were quantified. The study protocol was approved by Medical Ethics Committee of Zhongnan Hospital, Wuhan University (No. 2024071K), and complied with national regulations and international ethical standards for human research. Written informed consent was obtained from all participants prior to sample collection. Exclusion criteria included: (i) family history of genetic diseases; (ii) chronic diseases or conditions affecting cortisol levels or reproductive health; (iii) self‐reported history of smoking, alcohol abuse, or regular medication use; (iv) BMI outside the normal range (18.5–23.9 kg/m^2^); (v) any other clinical conditions deemed unsuitable for study participation by the attending physicians. This recruitment strategy was designed to minimize potential confounding from lifestyle and health‐related factors.

### 4.6 ELISA Quantification

ACTH (Cusabio Biotechnology, China), CORT (Cusabio Biotechnology, China), Glu (Nanjing Jiancheng, China), and α‐KG (Beyotime, China) were measured by ELISA according to manufacturers' protocols.

### 4.7 Behavioral Testing

Prior to behavioral assessments, animals underwent 1‐week acclimation to the testing room. All tests were conducted between 08:00‐18:00 daily.

OFT: The apparatus featured an open box with black interior walls. Rats were positioned at the arena center and allowed 5 min free exploration [72]. Exploratory trajectories and behavioral parameters were recorded using the SMART v3.0 video tracking system (Panlab, Spain).

EPM: The maze consisted of 2 open and 2 enclosed arms connected by a central platform. Surfaces were cleaned with 75% ethanol before each trial to eliminate odor cues. Rats were placed on the central platform for 5 min free exploration [73]. Activity was tracked using SMART v3.0.

NSFT: After 24 h food deprivation (water available), rats underwent a 5 min trial in an open field arena. A food‐containing dish was positioned centrally [74]. Latency to initial food bite was recorded via SMART v3.0, with increased latency indicating anxiety‐like behavior. To ensure recording stability, a 5 s stabilization period was set at the beginning of each trial; the final latency was calculated as the recorded time plus 5 s.

SPT: The 5 day protocol comprised:

Days 1–2: Individual housing with 2 bottles of 1% sucrose solution;

Day 3: One sucrose bottle replaced with water;

Day 4: 22 h food/water deprivation;

Day 5: 2 h test with simultaneous sucrose/water presentation (bottle positions alternated mid‐test).

Sucrose preference = [sucrose intake/(sucrose + water intake)] × 100%.

FST: Rats were placed in transparent cylinders (30 cm diameter × 60 cm height) containing 30 cm deep water (22–25°C). Immobility time during 5 min sessions was quantified by video tracking.

TST: Rats were suspended upside down for 6 min. Immobility duration during the final 4 min was recorded.

### 4.8 GLS and miRNA Intervention

Following anesthesia with 2% isoflurane, rats were immobilized in a stereotaxic frame after cranial depilation and ethanol disinfection. Head fixation was achieved using ear bars and an incisor adapter while maintaining unimpeded respiration. Following midline scalp incision and skull exposure, target coordinates were determined relative to bregma (5.6 mm posterior, 4.6 mm lateral, 7.4 mm ventral). Viral vectors were infused via Hamilton syringe at 120 nL/min, including: AAV‐hSyn‐rGLS‐WPRE (AAV9, 2.0 × 10^13^ GC/mL, VectorBuilder, China), AAV‐hSyn‐shGLS‐WPRE (AAV9, 2.0 × 10^13^ GC/mL, VectorBuilder, China), AAV‐hSyn‐miRNAs‐WPRE (AAV9, 2.0 × 10^13^ vg/mL, WZ biosciences, China), or AAV‐hSyn‐miRNAs_sponge‐WPRE (AAV9, 2.0 × 10^13^ vg/mL, WZ biosciences, China). Postinfusion, a 10 min diffusion period preceded needle withdrawal at identical infusion rate. Surgical sites were sutured and animals received postoperative care.

### 4.9 Neural Circuit Mapping with Viral Tracers

Stereotaxic brain injections were performed under anesthesia using a stereotactic frame (RWD, China). Viral suspensions (400–800 nL volume, adjusted for expression strength and titer) were infused through calibrated glass microelectrodes via an infusion pump at 120 nL/min. Postinfusion, pipettes remained in situ for 5–10 min to prevent reflux. Coordinates were defined relative to bregma: anterior‐posterior (AP), mediolateral (ML), and dorsal‐ventral (DV).

For monosynaptic anterograde tracing, control offspring rats received vCA1 injections (AP: −5.60 mm, ML: −4.60 mm, DV: −7.40 mm) of either AAV‐ CaMKIIα‐EGFP (AAV2/1, 1.0 × 10^12^ vg/mL, 800 nL) or AAV‐CaMKIIα‐Cre (AAV2/9, 1.0 × 10^12^ vg/mL, 800 nL). Concurrently, Pir received AAV‐DIO‐GAD67‐mCherry (AAV2/9, 1.0 × 10^12^ vg/mL, 600 nL; AP: +3.20 mm, ML: −2.70 mm, DV: −7.56 mm). Additional cohorts received Pir injections of AAV‐mDIX‐Cre (AAV2/9, 2.0 × 10^12^ vg/mL, 600 nL) or AAV‐mDIX‐EGFP (AAV2/1, 1.0 × 10^12^ vg/mL, 600 nL), with PVN injected with AAV‐CRH‐DIO‐mCherry (AAV2/9, 1.0 × 10^12^ vg/mL, 400 nL; AP: −0.9 mm, ML: −0.4 mm, DV: −7.80 mm). After 3 weeks, transcardial perfusion was performed under anesthesia, and brain sections were analyzed for EGFP/mCherry signals.

For retrograde monosynaptic tracing, PVN or Pir of control offspring were injected with helper viruses: AAV‐EF1α‐DIO‐mCherry‐F2A‐TVA (AAV2/8, 2.0 × 10^12^ vg/mL, 400 nL) and AAV‐EF1α‐DIO‐oRVG (AAV2/8, 2.0 × 10^12^ vg/mL, 400 nL), combined with Cre‐recombinase vectors: AAV‐CRH‐Cre (AAV2/9, 2.0 × 10^12^ vg/mL, 400 nL) or AAV‐mDIX‐Cre (AAV2/9, 2.0 × 10^12^ vg/mL, 400 nL). Three weeks later, EnvA‐pseudotyped RV‐ΔG‐EGFP rabies virus (2.0 × 10^12^ IFU/mL, 400 nL) was injected at identical coordinates. Perfusion and EGFP signal tracing followed 1 week postinjection.

For vCA1 → Pir → PVN triple tracing, vCA1 received AAV‐CaMKIIα‐Cre, Pir received AAV‐DIO‐mCherry (AAV2/9, 1.0 × 10^12^ vg/mL, 600 nL), and ipsilateral PVN received Cre‐dependent helper viruses plus AAV‐CRH‐Cre. RV was injected after 3 weeks. mCherry/EGFP signals were analyzed 1 week post‐RV injection.

For chemogenetic manipulation, Pir of PPCE offspring received AAV‐DIO‐hM3Dq‐EGFP (AAV2/9, 1.0 × 10^13^ vg/mL, 600 nL), while ipsilateral PVN received retro‐AAV‐Vgat1‐Cre (AAV2/R, 2.0 × 10^12^ vg/mL, 400 nL). After 3 weeks, animals underwent 2‐week ice‐water stress with intraperitoneal CNO (1 mg/kg) administered 1 h prestress. For circuit inhibition, vCA1 received AAV‐hSyn‐Cre (AAV2/1, 1.0 × 10^12^ vg/mL, 800 nL), PVN received retro‐AAV‐DIO‐hM3Dq‐mCherry (AAV2/Retro, 2.0 × 10^12^ vg/mL, 400 nL), and Pir received cannula implants in PPCE offspring. CNO/vehicle was microinjected via cannula 1 h prestress.

For optogenetics, vCA1 of control/PPCE/GLS‐treated offspring received AAV‐CaMKIIα‐ChR2‐EGFP (AAV2/9, 2.0 × 10^12^ vg/mL, 800 nL), Pir received AAV‐DIO‐Vgat1‐mCherry (AAV2/9, 2.0 × 10^12^ vg/mL, 600 nL), and PVN received retro‐AAV‐Cre (AAV2/R, 2.0 × 10^12^ vg/mL, 400 nL). Optophysiology commenced after 3 weeks. In separate cohorts, Pir received AAV‐Vgat2‐ChR2‐EGFP (AAV2/9, 2.0 × 10^12^ vg/mL, 600 nL) and PVN received AAV‐CRH‐mCherry (AAV2/9, 2.0 × 10^12^ vg/mL, 400 nL) in control/PPCE rats, followed by opto–electrophysiology after 3 weeks.

All viruses were sourced from BrainVTA (China). Postexperiment, transcardial perfusion with ice‐cold saline and 4% paraformaldehyde was performed. Animals with missed injections were excluded.

### 4.10 Electrophysiological Recordings in Brain Slices

Coronal sections (250–300 µm) containing Pir or PVN regions were prepared from adult offspring rats that had undergone stereotaxic viral injections ≥3 weeks prior. Sectioning was performed in oxygenated (95% O_2_/5% CO_2_), ice‐cold ACSF containing (in mm): 125 NaCl, 2.5 KCl, 1 MgCl_2_, 2 CaCl_2_, 1.25 NaH_2_PO_4_, 25 NaHCO_3_, and 11 D‐glucose. Slices were immediately transferred to an oxygenated ACSF incubation chamber maintained at 32–34°C for ≥1 h recovery before recording. Individual slices were positioned in a recording chamber mounted on an Olympus BX51WI upright microscope equipped with differential interference contrast optics and infrared visualization. Continuous perfusion (2 mL/min) with temperature‐regulated (32–34°C via Warner Instruments TC‐324B in‐line heater), oxygenated ACSF was maintained. Whole‐cell voltage‐clamp recordings were obtained from neurons exhibiting maximal ChR2‐EGFP‐positive axonal density using patch pipettes (3–5 MΩ) filled with: voltage‐clamp internal solution: 135 CsMeSO_3_, 10 HEPES, 1 EGTA, 3.3 QX‐314, 4 Mg‐ATP, 0.3 Na_2_‐GTP, and 8 Na_2_‐phosphoCreatine (pH 7.3 adjusted with CsOH, 295 mOsm); current‐clamp internal solution: 145 KGlu, 10 HEPES, 0.2 EGTA, 1 MgCl_2_, 4 Mg‐ATP, 0.3 Na_2_‐GTP, and 10 Na_2_‐phosphoCreatine (pH 7.3 adjusted with KOH, 295 mOsm). All recordings compensated for 10 mV liquid junction potential. ChR2 was activated using 473 nm laser light (Opto Engine) focused via optical fiber onto recorded neurons. Light pulses (1–2 mW/mm^2^, 473 nm) were delivered at 15 s or 200 ms intervals using acquisition software. During voltage‐clamp recordings of optically evoked synaptic currents, 0.5 µm TTX and 100 µm 4‐AP were added to perfusate to block action potentials and suppress network activity.

### 4.11 Whole Transcriptome Sequencing

Total RNA was extracted from male rat vCA1 tissues using TRIzol reagent (Invitrogen, USA). Sequencing libraries were prepared with the TruSeq Stranded Total RNA Library Prep Kit (Illumina, USA). Quality control and bioinformatic analyses were conducted via the Majorbio Cloud Platform (Shanghai Majorbio Bio‐pharm Technology Co., Ltd, China).

### 4.12 WGBS

WGBS was conducted by Novogene (China) using standard Illumina protocols. Briefly, adaptors were ligated to sonicated DNA fragments, followed by bisulfite conversion per manufacturer specifications. Libraries were prepared from 100 ng bisulfite‐treated DNA and sequenced on an Illumina HiSeq 2000 system (100‐bp paired‐end reads). Each sample included a 5% PhiX genomic DNA spike‐in control. Methylation patterns were visualized with IGV.

### 4.13 BSP

BSP analysis was performed by G&C Biotechnology (China). Genomic DNA was extracted using commercial kits, subjected to bisulfite treatment, and amplified via PCR with target‐specific primers. PCR products were sequenced to determine site‐specific methylation status.

### 4.14 Luciferase Reporter Assay

H19‐7 cells were cotransfected with luciferase reporter plasmids containing either WT or MUT GLS 3′UTR binding sites, along with miRNAs mimic or miRNAs negative control (miRNA NC). All constructs were synthesized by GenePharma Co. (China). At 48 h post‐transfection, luciferase activity was quantified using the Dual‐Luciferase Reporter Assay Kit and a GloMax 20/20 luminometer.

### 4.15 Real‐Time Quantitative PCR

Total RNA was extracted from rat vCA1 tissue using TRIzol (Invitrogen, USA). cDNA synthesis utilized HiScript II Select qRT SuperMix (Vazyme, China) for mRNAs or miRNA Reverse Transcription Kit (QIAGEN Bio, Germany) for miRNAs. All primers (Table S1) were synthesized by Sangon Biotech. Relative expression was calculated via the 2^−ΔΔct^ method normalized to *glyceraldehyde‐3‐phosphate dehydrogenase* (*Gapdh*) or *RNU6A* (*U6*) gene.

### 4.16 Western Blot Analysis

Total protein was extracted from rat vCA1 and PVN tissues using RIPA lysis buffer supplemented with 1 mm PMSF. Proteins were separated by 10% sodium dodecyl sulfate‐polyacrylamide gel electrophoresis (SDS‐PAGE) and transferred to PVDF membranes (Millipore, USA). Membranes were blocked with 5% non‐fat milk and incubated overnight at 4°C with primary antibodies: rabbit anti‐CRH (1:100, ABclonal, China), rabbit anti‐GLS (1:100, ABclonal, China), rabbit anti‐vGluT1 (1:1000, Abcam, USA), and mouse anti‐GAPDH (1:2000, ABclonal, China). After washing, membranes were incubated with HRP‐conjugated secondary antibodies (goat anti‐rabbit/mouse IgG) for 2 h at room temperature. Protein bands were visualized using ECL substrate (Tanon, China) and quantified with ImageJ (v6.0).

### 4.17 Immunofluorescence Staining

Perfusion‐fixed brain tissues were cryoprotected in graded sucrose solutions (20%, 25%, 30%), embedded in OCT compound (Sakura Finetek, USA), and sectioned coronally (10 µm). After antigen retrieval and blocking with 10% goat serum, sections were incubated overnight at 4°C with primary antibodies: rabbit anti‐Glu (1:500, Abcam, USA), rabbit anti‐c‐Fos (1:200, ABclonal, China), and goat anti‐GAD67 (1:200, R&D Systems, USA). Secondary antibody incubation (90 min) was followed by DAPI counterstaining. Images were acquired using an Olympus fluorescence microscope and quantified with ImageJ.

### 4.18 Statistical Analysis

Data are expressed as mean ± SEM. Analyses were performed using GraphPad Prism 9.0 (GraphPad Software, USA) with experimenters blinded to groups. Two‐group comparisons used unpaired two‐tailed *t*‐test, Welch's *t* test or Mann Whitney test. Multigroup comparisons employed Friedman test with Dunn's multiple comparison post hoc test, one‐way ANOVA with Tukey's multiple comparison post hoc test or two‐way ANOVA with Sidak's multiple comparison post hoc test. *p* < 0.05 defined statistical significance.

## Ethics Statement

The Animal Study Protocol was approved by the Animal Care and Use Committee (ACUC) of Wuhan University, China (Permit No. JC2020‐015). The study Adhered to the Guidelines Set By the Committee.

## Conflicts of Interest

The authors declare no conflicts of interests.

## Supporting information




**Supporting File**: advs75380‐sup‐0001‐SuppMat.docx.

## Data Availability

The data that support the findings of this study are available from the corresponding author upon reasonable request.
