# Supplementary material for: Paternal Caffeine Exposure Programs Offspring Stress Vulnerability via Sperm Dlk1‐Dio3 Imprinting‐Directed Remodeling of a Novel Neural Circuit
Source: Adv Sci (Weinh). 2026 Apr 30;13(40):e75380. doi: 10.1002/advs.75380 (PMC13335588; doi:10.1002/advs.75380)
Supplement: Supplementary file 1 — Supporting File: advs75380‐sup‐0001‐SuppMat.docx. [file ADVS-13-e75380-s001.docx]

Supplementary Materials for

Paternal Caffeine Exposure Programs Offspring Stress Vulnerability *via* Sperm Dlk1-Dio3 Imprinting-Directed Remodeling of a Novel Neural Circuit

**Authors:** Mengxi Lu^1 #^, Gaole Dai^1 #^, Sen Zhu^1 #^, Shuai Zhang^1^, Tingting Wang^1^, Yuan Meng^1^, Fang Yang^1^, Xiaoyi Han^1^, Hui Wang^2, 3^, Hao Kou ^1, 3 *^, Dan Xu^1, 3, 4 *^

^#^These authors contributed equally.

*****Corresponding author: Dan Xu (xuyidan70188@whu.edu.cn), Hao Kou (kouhao007@whu.edu.cn)

**This file includes:**

Figures. S1 to S8

Table S1

Figure. S1.

**
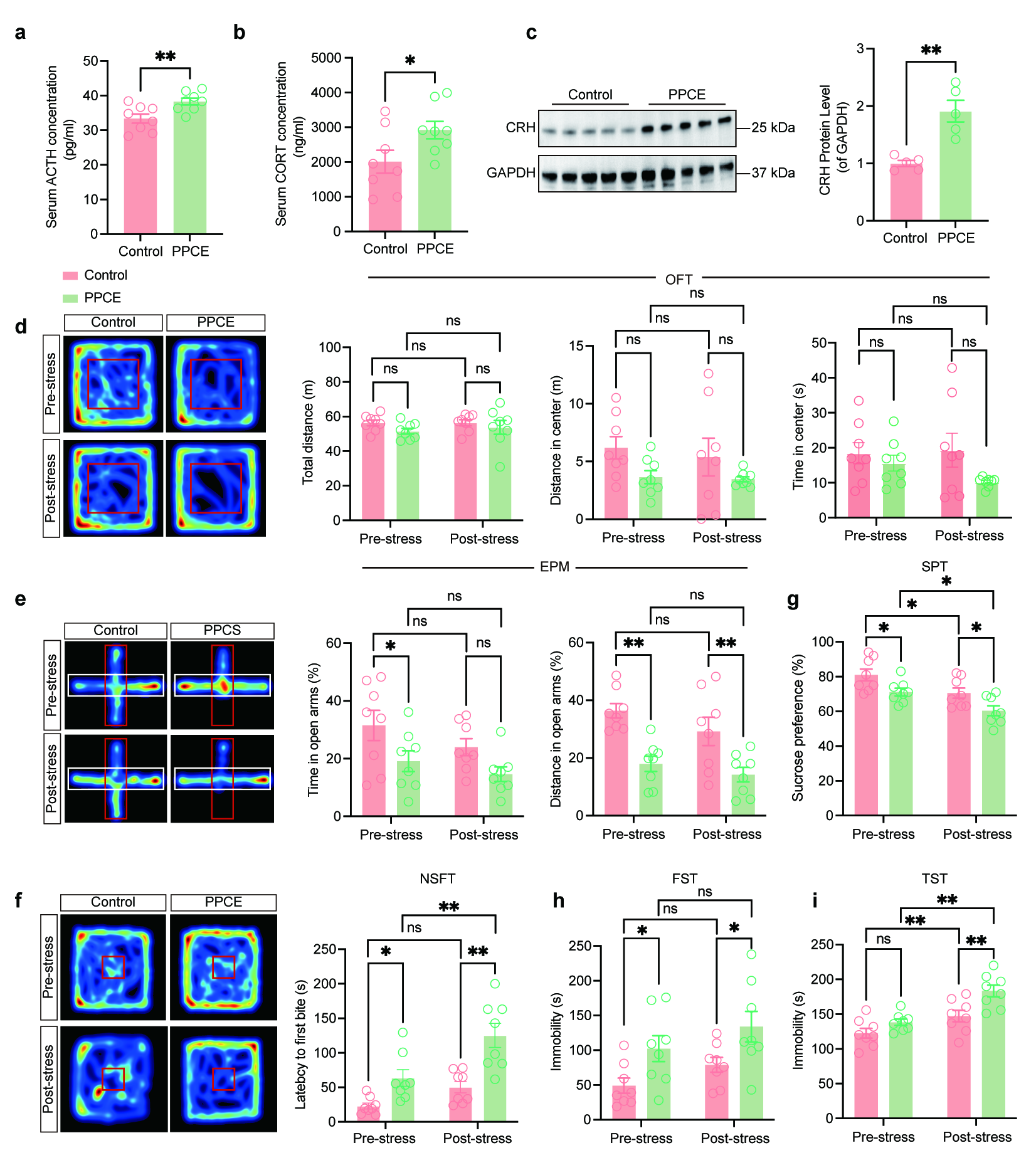
**

**Supplementary Fig. 1 PPCE female offspring display sex-dimorphic stress phenotypes.** (**a**) Serum ACTH levels in female PPCE offspring rats (n = 8 rats per group). (**b**) Serum CORT levels in female PPCE offspring rats (n = 8 rats per group). (**c**) CRH protein expression level in female PPCE offspring rats (n = 5 rats per group). (**d**) Schematic diagram of representative rat activity trajectories and statistical indicators in the OFT, including total distance, distance in center and time in center (n = 8 rats per group). (**e**) Schematic diagram of representative rat activity trajectories and statistical indicators in the EPM test, including time and distance spent in open arms (n = 8 rats per group). (**f**) Schematic diagram of representative rat activity trajectories and latency to first bite statistics in the NSFT (n = 8 rats per group). (**g**) Sucrose preference index in the SPT (n = 8 rats per group). (**h**) Immobility time in the FST (n = 8 rats per group). (**i**) Immobility time in the TST (n = 8 rats per group). Data are presented as mean ± SEM. ns, not significant; *^*^P* < 0.05, *^**^P* < 0.01; by unpaired two-tailed t-test (a, b), Welch's t test (c), or two-way ANOVA (d-i).

Figure. S2.

**
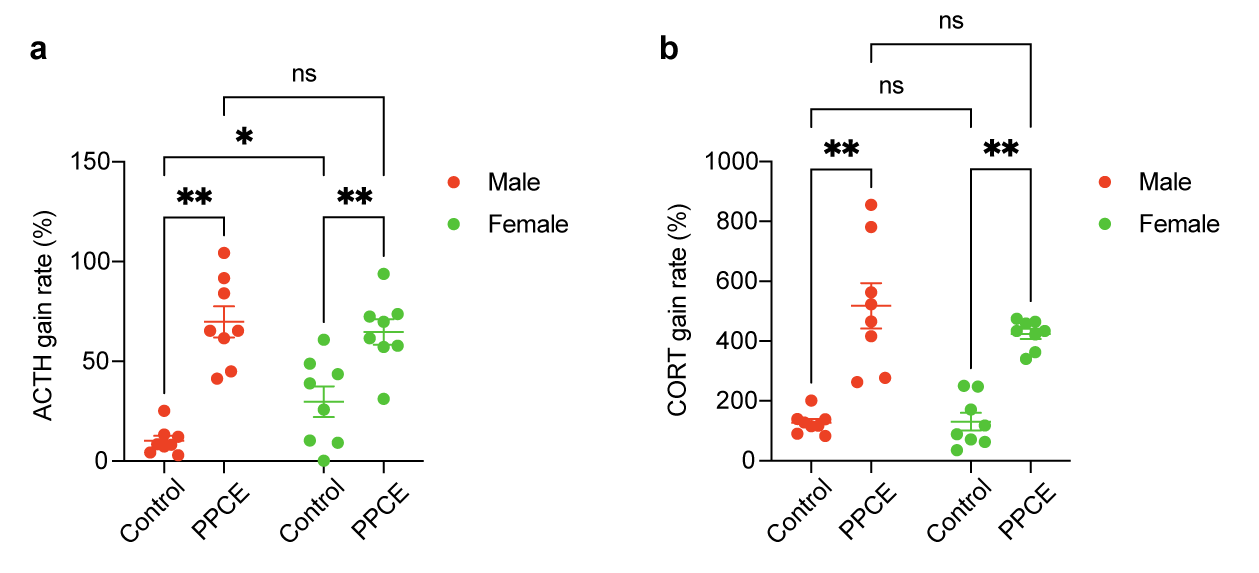
**

**Supplementary Fig. 2 Effect of PPCE on the gain rate of stress hormones.** (**a**) ACTH gain rate. (**b**) CORT gain rate. Gain rate was calculated as: [(post-stress level - baseline level) / baseline level] × 100%. Data are presented as mean ± SEM. ns, not significant; *^*^P* < 0.05, *^**^P* < 0.01; by two-way ANOVA (a, b).

Figure. S3.

**
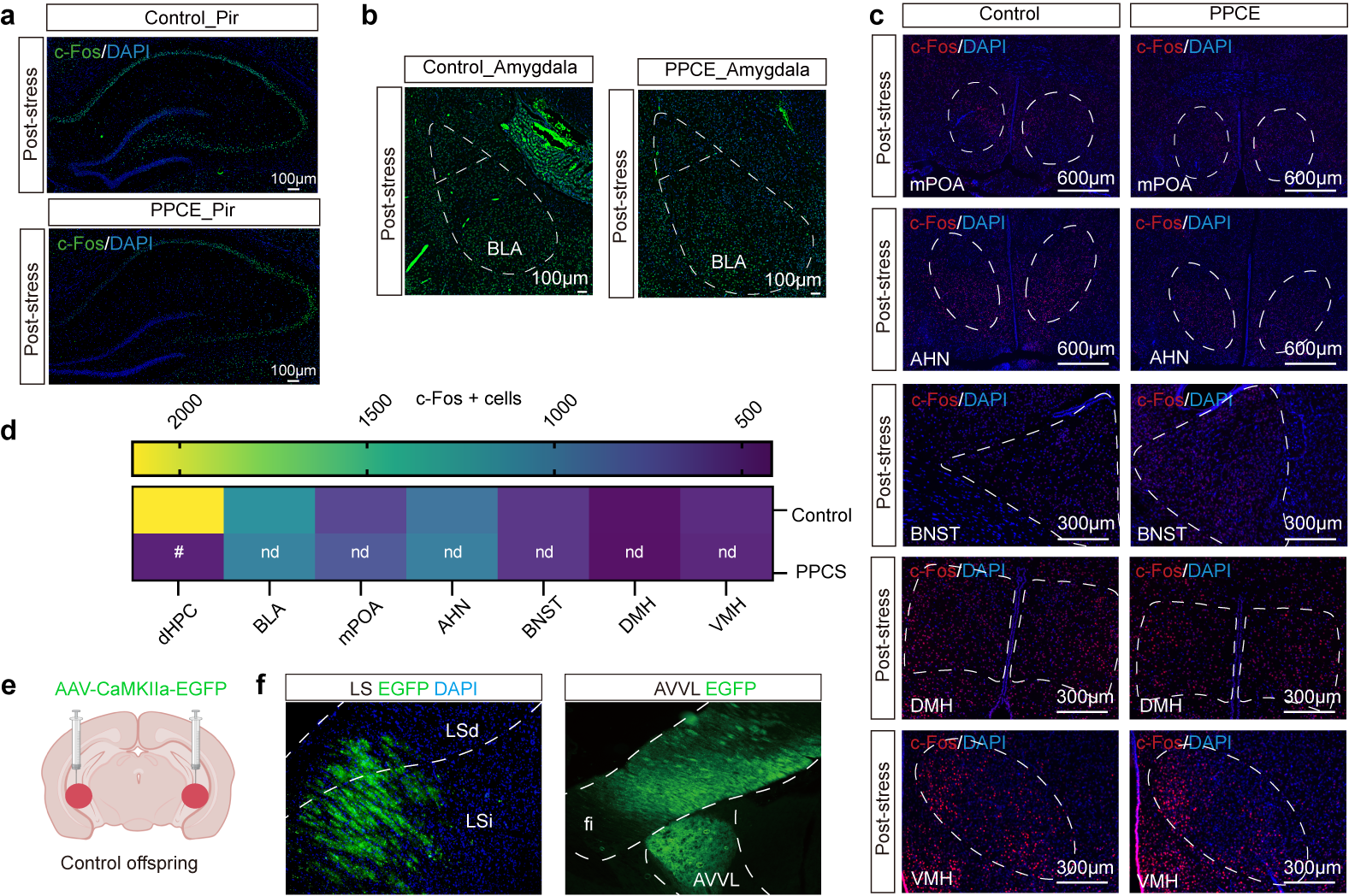
**

**Supplementary Fig.** **3 Region-specific c-Fos alterations in HPA regulatory nuclei.** (**a-d**) c-Fos immunofluorescence staining and positive cell count statistics (n = 3 rats per group; dHPC: *q* = 0.005979, BLA: *q* = 0.800114, mPOA: *q* = 0.800114, AHN: *q* = 0.800114, BNST: *q* = 0.800114, DMH: *q* = 0.800114, VMH: *q* = 0.800114). (**e**) Viral injection strategy. (**f**) Representative images of EGFP-positive signals expressed in LS and AVVL. nd, no discovery; *^#^q* < 0.01; by multiple unpaired t-test (d).

Figure. S4.


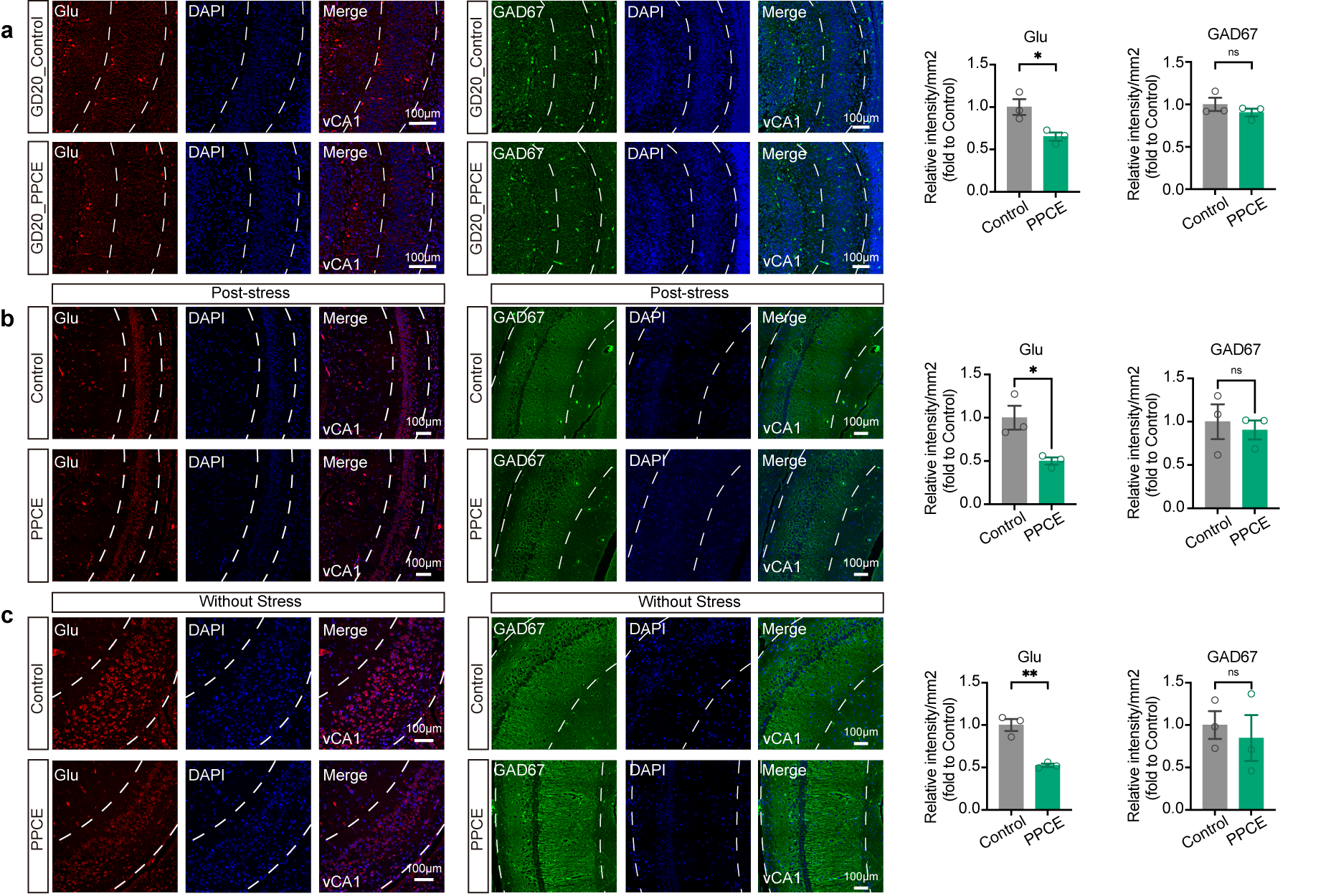


**Supplementary Fig. 4 vCA1 glutamatergic neuron impairment in PPCE offspring.** (**a**) Immunofluorescence staining of Glu (red) and GAD67 (green) and statistical analysis of fluorescence intensity (n = 3 rats per group). (**b**) Immunofluorescence staining of Glu (red) and GAD67 (green) and statistical analysis of fluorescence intensity (n = 3 rats per group). (**c**) Immunofluorescence staining of Glu (red) and GAD67 (green) and statistical analysis of fluorescence intensity (n = 3 rats per group). Data are presented as mean ± SEM. ns, not significant; *^*^P* < 0.05, *^**^P* < 0.01; by unpaired two-tailed t-test (a-c).

Figure. S5.


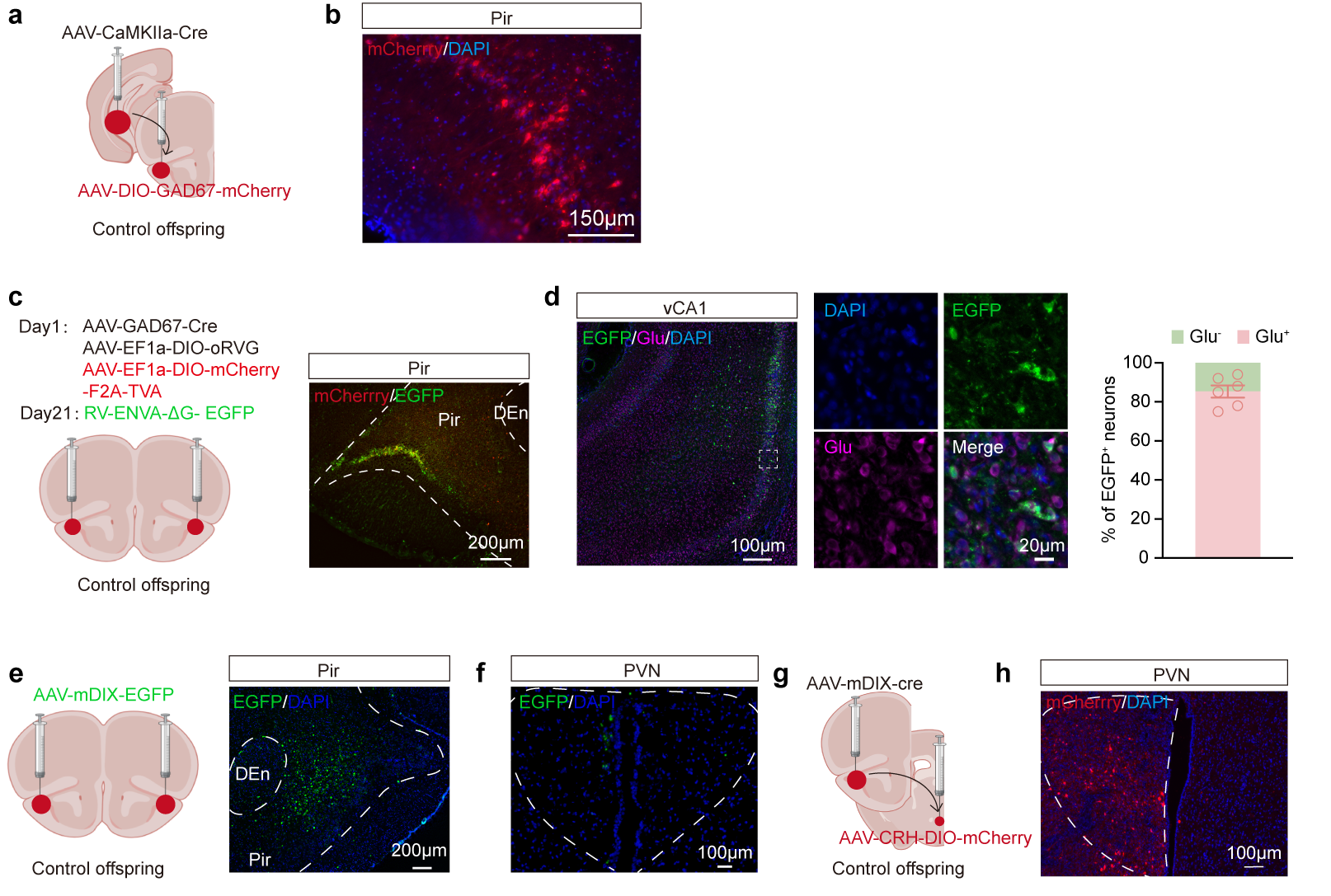


**Supplementary Fig. 5 Neuroanatomical validation of vCA1^Glu^ → Pir^GABA^ → PVN^CRH^ circuit.** (**a** and **b**) Viral injection strategy and representative images of mCherry-positive signals expressed in Pir. (**c** and **e**) Viral injection strategy and fluorescent representation of injection site. (**d**) Representative images of EGFP-positive signal in vCA1 (left) and EGFP-labeled neurons co-localized with Glu antibody within vCA1 (right). (**f**) Representative images of EGFP-positive signals expressed in PVN. (**g** and **h**) Viral injection strategy and representative images of mCherry-positive signals expressed in Pir. Data are presented as mean ± SEM.

Figure. S6.

**
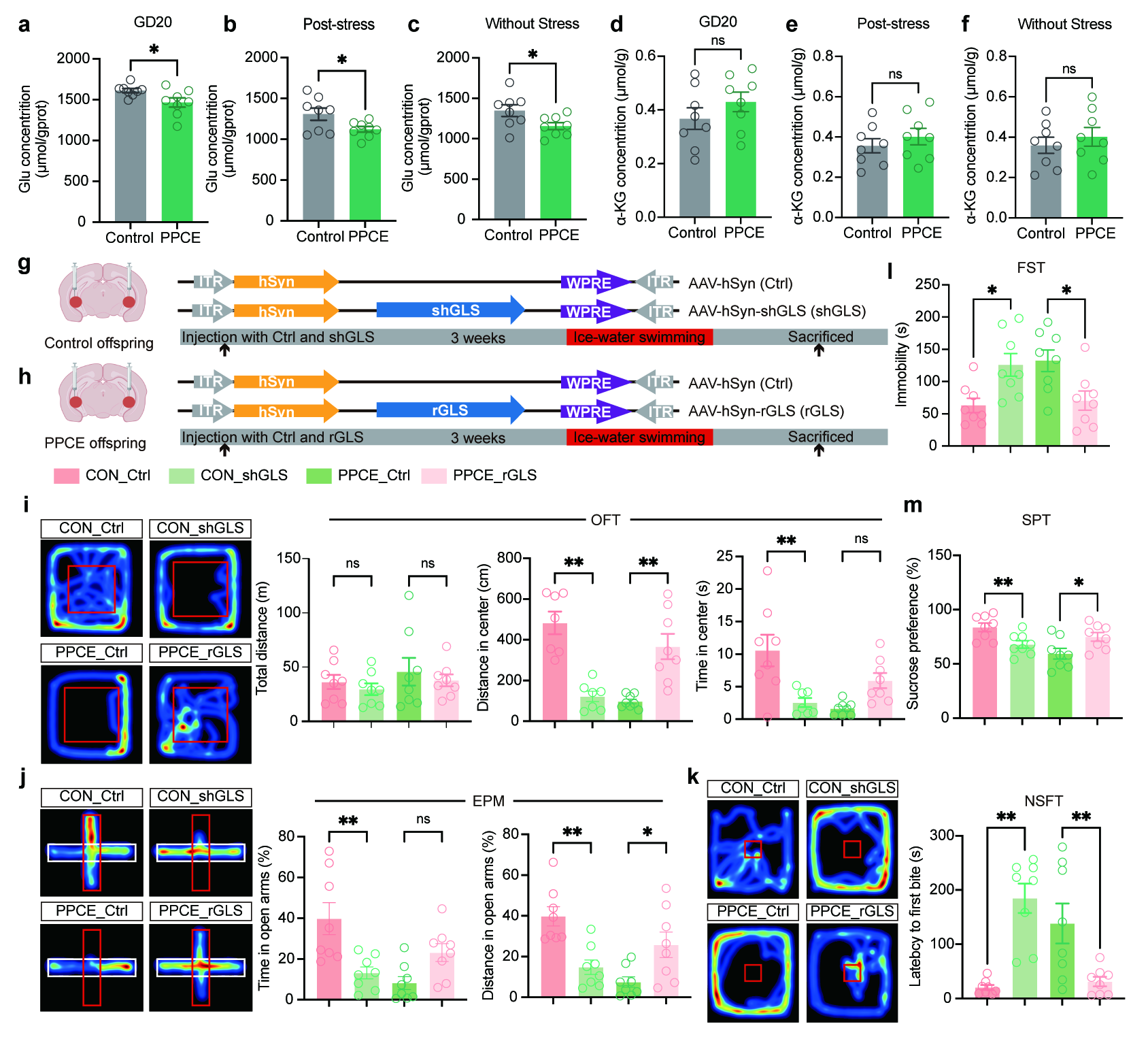
**

**Supplementary Fig. 6 GLS suppression mediates affective behaviors.** (**a-c**) Glu concentration in the vCA1 tissue of PPCE offspring rats (n = 8 litters or rats per group). (**d-f**) α-KG concentration in the vCA1 tissue of PPCE offspring rats (n = 8 litters or rats per group). (**g** and **h**) Schematic diagram of viral injection and animal handling. (**i**) Schematic diagram of representative rat activity trajectories and statistical indicators in the OFT, including total distance, distance in center and time in center (n = 7 or 8 rats per group). (**j**) Schematic diagram of representative rat activity trajectories and statistical indicators in the EPM test, including time and distance spent in open arms (n = 8 rats per group). (**k**) Schematic diagram of representative rat activity trajectories and latency to first bite statistics in the NSFT (n = 8 rats per group). (**l**) Immobility time in the FST (n = 8 rats per group). (**m**) Sucrose preference index in the SPT (n = 8 rats per group). Data are presented as mean ± SEM. ns, not significant; *^*^P* < 0.05, *^**^P* < 0.01; by unpaired two-tailed t-test (a-f), Welch's t test (a) or one-way ANOVA (i-m).

Figure. S7.

**
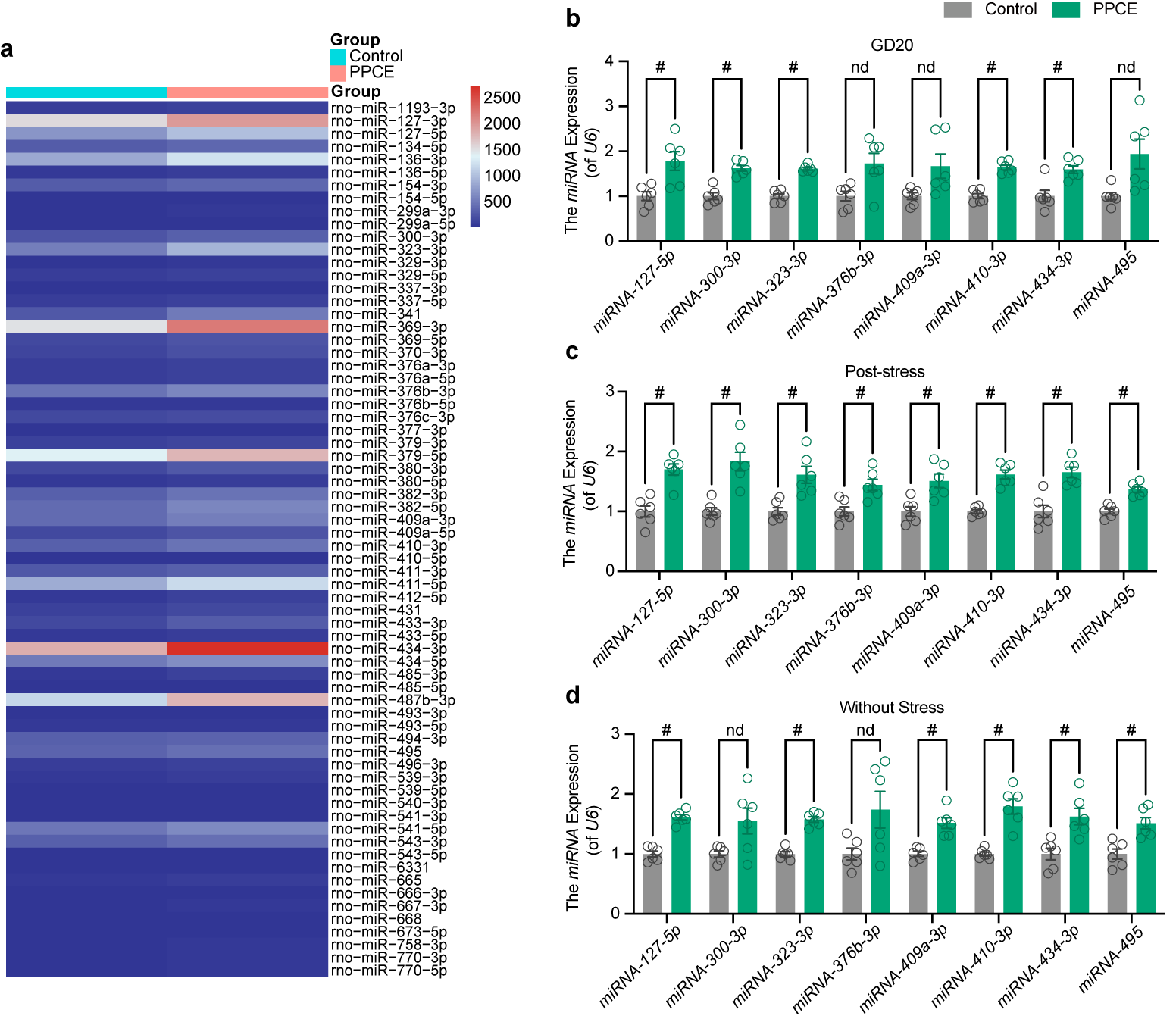
**

**Supplementary Fig. 7 Persistent Dlk1-Dio3 miRNA upregulation in vCA1.** (**a**) Heatmap of miRNA expression in PPCE offspring vCA1 tissue. (**b**) *miRNA* expression level in GD20 PPCE offspring rats (n = 6 litters per group). (**c**) *miRNA* expression level in post-stress PPCE offspring rats (n = 6 rats per group). (**d**) *miRNA* expression level in without-stress PPCE offspring rats (n = 6 rats per group). Data are presented as mean ± SEM. nd, no discovery; *^#^q* < 0.01; by multiple unpaired t-test (b-d).

Figure. S8.

**
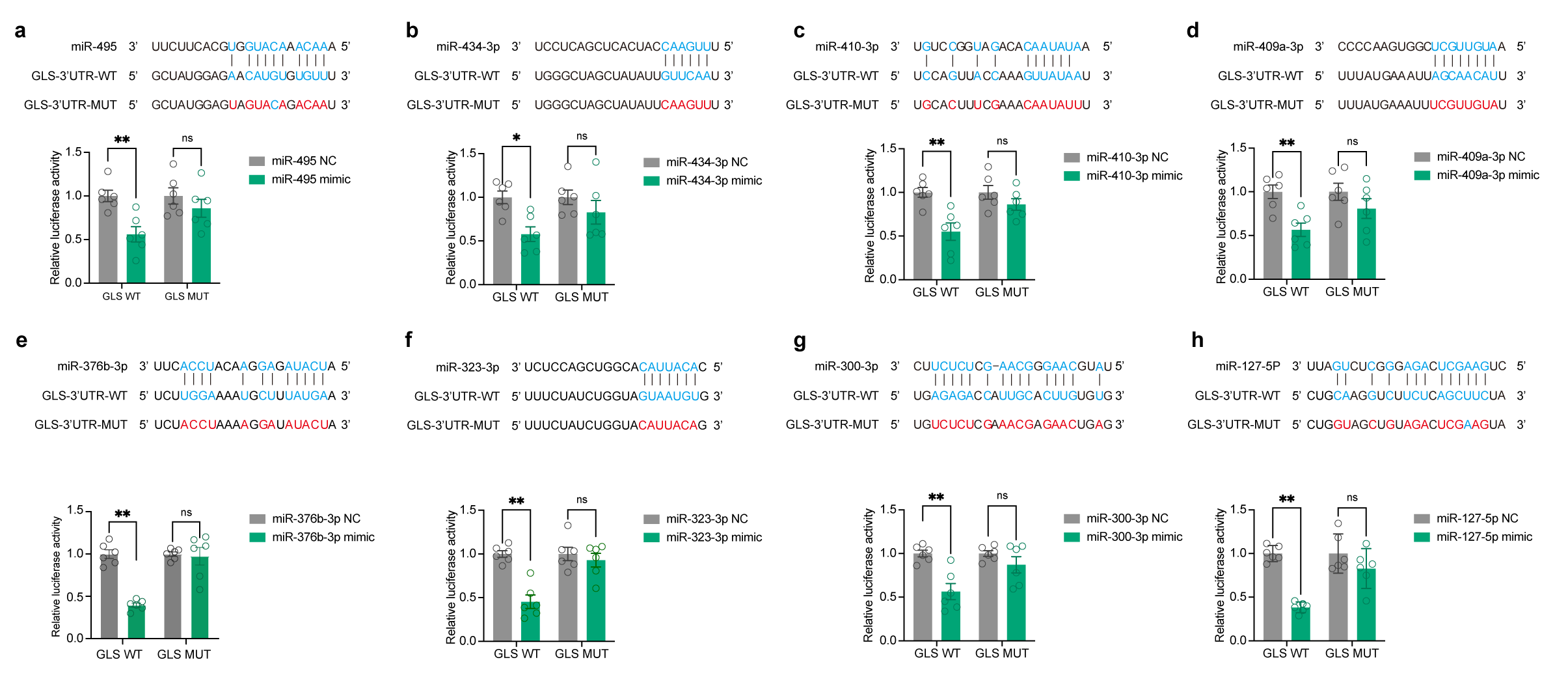
**

**Supplementary Fig. 8** **Dlk1-Dio3 miRNAs directly target GLS 3'UTR.** (**a-h**) Diagram of predicted miRNA binding sites and corresponding MUT sites in the 3'UTR of GLS mRNA, and dual-luciferase reporter gene assay to confirm the direct targeting of miRNAs to GLS in H19-7 cell line (n = 6). Data are presented as mean ± SEM. ns, not significant; *^*^P* < 0.05, *^**^P* < 0.01; by two-way ANOVA (a-h).

Table S1. Oligonucleotide primers used in RT-qPCR analysis

| Genes | Forward primers | Reverse primers |
| --- | --- | --- |
| *Gls* | AGGTGGTGATCAAAGGGTAAAG | TCCATGTCCATAGCTGACAAAG |
| *Gapdh* | ACTCCCATTCTTCCACCTTTG | CCCTGTTGCTGTAGCCATATT |
| *rno-miR-127-5p* | CGCTGAAGCTCAGAGGGCT | miScript Universal Primer |
| *rno-miR-300-3p* | GCGTATGCAAGGGCAAGCT | miScript Universal Primer |
| *rno-miR-323-3p* | GCGCACATTACACGGTCG | miScript Universal Primer |
| *rno-miR-376b-3p* | CGCGCGATCATAGAGGAACAT | miScript Universal Primer |
| *rno-miR-409a-3p* | CGCGAATGTTGCTCGGTG | miScript Universal Primer |
| *rno-miR-410-3p* | CGCGCGAATATAACACAGATG | miScript Universal Primer |
| *rno-miR-434-3p* | CGCGTTTGAACCATCACTCG | miScript Universal Primer |
| *rno-miR-495* | GCGAAACAAACATGGTGCA | miScript Universal Primer |
| *mmu-miR-127-5p* | CGCTGAAGCTCAGAGGGC | miScript Universal Primer |
| *mmu-miR-300-3p* | GCGTATGCAAGGGCAAGCT | miScript Universal Primer |
| *mmu-miR-323-3p* | GCGCACATTACACGGTCG | miScript Universal Primer |
| *mmu-miR-376b-3p* | CGCGCGATCATAGAGGAACAT | miScript Universal Primer |
| *mmu-miR-410-3p* | CGCGCGAATATAACACAGATG | miScript Universal Primer |
| *mmu-miR-434-3p* | CGCGTTTGAACCATCACTCG | miScript Universal Primer |
